# CuI nanoparticles supported on a novel polymer-layered double hydroxide nanocomposite: an efficient heterogeneous nanocatalyst for the synthesis of bis-*N*-arylsulfonamides†

**DOI:** 10.1039/d1ra02086b

**Published:** 2021-05-26

**Authors:** Jamshid Babamoradi, Ramin Ghorbani-Vaghei, Sedigheh Alavinia

**Affiliations:** Department of Chemistry, Bu-Ali Sina University Zip Code 65174 Hamedan Iran rgvaghei@yahoo.com +98-81-38380647

## Abstract

A new type of polymer-layered double hydroxide nanocomposite bearing thiazole moieties was used to support CuI nanoparticles (NPs) as a heterogeneous catalyst for the synthesis of bis-*N*-arylsulfonamides. The prepared nanostructured catalyst (LDH@MPS-GMA-TZ-CuI) showed high catalytic activity, as well as excellent recyclability for the preparation of bis-*N*-arylsulfonamides *via* the chemoselective reaction of 1,3-disulfonyl chloride and nitroarenes. The superior catalytic activity of the LDH@MPS-GMA-TZ-CuI is related to the high loading of CuI NPs and favorable surface properties.

## Abstract 2

CuI NPs supported on novel polymer–LDHs nanocomposite was investigated for the synthesis of bis-*N*-arylsulfonamides.

## Graphical abstract

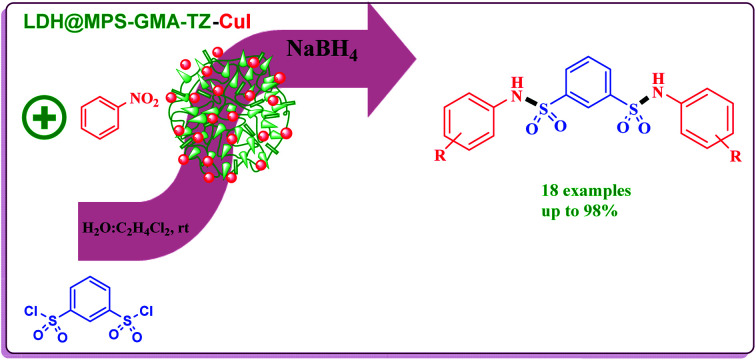

## 1. Introduction

Sulfonamide is a wonderful compound that has a clear biological effect, as it is used in the treatment of various diseases. Several studies in the field of medical chemistry have proven that sulfonamide derivatives possess a range of beneficial biological activities, being antimicrobial, antiviral, antitubercular, and anticancer agents.^1–3^ Although the amination of arylsulfonyl chlorides is still a valuable method for the synthesis of *N*-arylsulfonamides, the mentioned reactions typically require the use of strong bases that can negatively effect the recovery of the catalyst.^4^ Recently, transition metal-catalyzed carbon–nitrogen bond forming *via* cross-coupling reactions has represented a powerful means for the preparation of diverse sulfonamide compounds, which have high utilities in pharmaceutical, materials and chemical science.^5^ However, some of them require harsh reaction conditions and expensive noble and precious metal-based catalyst systems, which can increase the production costs due to difficult separation of the desired value-added products. Thus, it is of great significance to develop a new catalyst system capable of providing functionalized *N*-aryl sulfonamides with excellent yields under mild conditions. In this regard, the reaction of nitroarenes and sulfonyl chloride has been proven to be a practical method in the synthesis of *N*-arylsulfonamides. Hence, some research has been recently reported to synthesis of *N*-aryl sulfonamide derivatives from nitroarenes, such as combination of FeCl_2_ and *trans-N*,*N*′-dimethyl-1,2-diaminocyclohexane (DMDACH),^6^ Fe dust as a reducing agent,^7^ iron powder,^8^ iron-based metal–organic framework,^9^ insertion of sulfur dioxide,^10^ and electrochemical synthesis.^11^ However, despite recent progress, it is still a big challenge to design new heterogeneous support, focusing on reducing the use of expensive or homogeneous catalysts to achieve mild reaction conditions.

In this sense, layered double hydroxides (LDHs) with the formula [M^2+^ (1 − *x*) M*x*^3+^(OH)_2_] (A^*n*−^) *x*/*n z*H_2_O] have been widely investigated as heterogeneous support because of their low cost, ease of accessibility, large surface area, non-toxicity, recyclability and high stability.^12^ However, low loading problem has a negative effect on the catalytic activity of heterogeneous supports. In order to overcome this limitation, the functionalization of LDH; for example, post-synthesis, grafting and polymerization are particularly important methods to affect the catalytic activity of LDHs.^13^ Herein, we can refer to some specific methods applied to improve the catalytic performance of Cu–Zn–Al LDH support: (1) radical polymerization of glycidyl methacrylate (GMA) linkage, (2) presenting thiazole moieties in order to decrease the aggregation of CuI NPs, (3) and immobilization of CuI NPs.

**Scheme 1:**
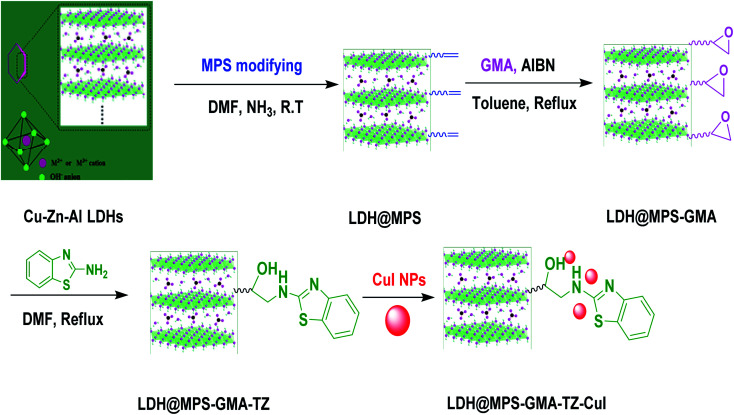
Schematic representation for preparation of LDH@MPS-GMA-TZ-CuI nanocomposite.

**Scheme 2:**
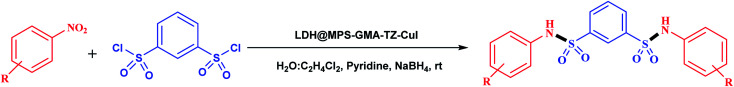
Catalytic efficiency of LDH@MPS-GMA-TZ-CuI in the synthesis of bis-*N*-arylsulfonamides.

Following our recent aim of designing multifunctional catalyst;^14^ in this article, we synthesized a LDH@MPS-GMA-TZ as a novel support with a thiazole moieties. After immobilization of CuI NPs, we aimed at analyzing the catalytic performance of LDH@MPS-GMA-TZ-CuI (Scheme 1) for the generation of bis-*N*-aryl sulfonamides from the reaction of 1,3-disulfonylchloride and nitroarenes with high yields (Scheme 2). The prepared nanocomposite revealed excellent catalytic function in the synthesis of different bis-*N*-aryl sulfonamides.

## 2. Experimental

### 2.1. Synthesis of LDH

Cu–Zn–Al-LDH was synthesized through the coprecipitation method as per the previously reported procedure.^15^ In a typical procedure, solution A was prepared by dissolving Zn(NO_3_)_2_·6H_2_O, Al(NO_3_)_2_·9H_2_O and Cu(NO_3_)_3_·6H_2_O in a ratio of 1 : 1 : 1 in deionized water (100 mL). Solution B was also prepared by dissolving Na_2_CO_3_ and NaOH in 100 mL of deionized water in order to form a mixed base solution. Then, the solution B was slowly added to the solution A at a pH value of 12. Then, the reaction – without being stirred – was set at 80 °C for 6 h. The solid compound obtained was separated by filtration then washed with distilled water and dried under vacuum conditions at 80 °C for 12 h. The synthesized Cu–Zn–Al-LDH was calcined in static air at 600 °C for 4 h (Scheme 1).

### 2.2. Preparation of silica coated LDH (LDH@MPS)

At first, Cu–Zn–Al-LDH (0.2 g) was dispersed in of DMF (10 mL) for 5 min in an ultrasonic cleaning apparatus. After sonication, ammonia solution (2 mL of 25 wt% solution) and 3-(trimethoxysilyl) propyl methacrylate (2 mL) were added carefully in to a round bottom flask. The resulting suspension was stirred at 50 °C for 24 h. The resultant suspension was filtered out and washed with ethanol (20 mL) and water (40 mL) and, then, dried at 50 °C (Scheme 1).^16^

### 2.3. Preparation of LDH coated with glycidyl methacrylate (LDH@MPS-GMA)

The LDH@MPS-GMA were synthesized through free radical polymerization method. To synthesize the LDH@MPS-GMA, the obtained LDH@MPS (0.2 g) was dispersed in 30 mL of toluene for 30 min, and then glycidyl methacrylate (2 mL) and azobisisobutyronitrile (0.25 g) were added to the above mixture and refluxed for 24 h. The functionalized LDH@MPS-GMA were filtered off and washed with toluene (20 mL), and dried under vacuum conditions at room temperature for 12 h (Scheme 1).^16^

### 2.4. Synthesis of LDH@MPS-GMA-TZ

For synthesis of LDH@MPS-GMA-TZ, the prepared LDH@MPS-GMA (0.2 g) was sonicated in DMF (50 mL) for 15 min. Afterwards, to the reaction solution, 2-aminobenzothiazole (0.6 g) was slowly added and, then, it was refluxed for 24 h. Subsequently, the LDH@MPS-GMA-TZ precipitate were collected, washed with DMF, and dried under vacuum (Scheme 1).

### 2.5. Procedure for the preparation of LDH@MPS-GMA-TZ-CuI

The CuI NPs were synthesized through our previously work.^14*c*^ The prepared support (0.1 g) and CuI NPs (0.05 g) were dispersed in 100 mL of water and then stirred for 5 h at room temperature. Subsequently, the LDH@MPS-GMA-TZ-CuI nanocomposite was separated through centrifusion, washed with water, and dried under vacuum. The ICP report indicated that the amount of copper in the final catalyst is 20.3%.

### 2.6. General procedure for the synthesis of bis-*N*-aryl sulfonamide derivatives (3a-o)

At first, the catalyst (50 mg) was dispersed in H_2_O : C_2_H_4_Cl_2_ (1 : 1) (2 mL) by sonication for 10 min and then nitroarene (0.5 mmol), 1,3-disulfonyl chloride (0.25 mmol), pyridine (0.5 mmol) and NaBH_4_ (1.0 mmol) was added to the mixture and stirred untill completion of the reaction monitored by TLC (*n*-hexane/ethyl acetate, 8 : 2). After the completion of the reaction, the reaction mixture was diluted with EtOAc (5 mL) and the LDH@MPS-GMA-TZ-CuI was separated by centrifuging and washed with ethanol (20 mL). The pure product was obtained by extraction with ethyl acetate (3 × 5 mL). The organic solution was dried by anhydrous sodium sulfate and, the solvent evaporated. Finally, the product was washed three times with HCl solution (10 mL, 0.1 M) and dried. In some products, the solid formed was filtered, dried and recrystallized from ethanol (10 mL) as yellow crystals.

### 2.7. Spectral data of compounds

#### *N* ^1^,*N*^3^-Di-*p*-tolylbenzene-1,3-disulfonamide

Mp 282–284 °C, ^1^H NMR (250 MHz, DMSO-*d*_6_) *δ* 10.32 (s, 2H), 8.36–7.47 (m, 4H), 6.92 (d, *J* = 18.0 Hz, 8H), 2.15 (s, 6H). ^13^C NMR (63 MHz, DMSO-*d*_6_) *δ* 143.74, 138.33, 138.02, 133.41, 131.17, 130.53, 129.23, 128.91, 128.34, 127.91, 124.65, 18.99. MS *m*/*z*: 416.

#### *N* ^1^,*N*^3^-Bis(4-methoxyphenyl)benzene-1,3-disulfonamide

Mp 269–270 °C, ^1^H NMR (250 MHz, DMSO-*d*_6_) *δ* 10.12 (s, 1H), 8.07 (s, 1H), 7.81 (d, *J* = 7.8 Hz, 1H), 7.65 (t, *J* = 7.9 Hz, 1H), 6.87 (d, *J* = 8.5 Hz, 2H), 6.75 (d, *J* = 8.7 Hz, 2H), 3.72 (s, 3H). ^13^C NMR (63 MHz, DMSO-*d*_6_) *δ* 157.33, 140.86, 131.08, 130.89, 129.71, 125.50, 124.46, 114.80, 55.58. MS *m*/*z*: 448.

#### *N* ^1^,*N*^3^-Bis(2,3-dimethoxyphenyl)benzene-1,3-disulfonamide

Mp 279–281 °C; ^1^H NMR (250 MHz, DMSO-*d*_6_) *δ* 9.57 (s, 1H), 8.90 (d, *J* = 5.4 Hz, 1H), 8.20–7.83 (m, 1H), 7.70 (dd, *J* = 7.8 Hz, 1H), 7.02 (d, *J* = 8.4 Hz, 1H), 6.68–6.15 (m, 2H), 3.68 (s, 4H), 3.26 (s, 3H). ^13^C NMR (63 MHz, DMSO-*d*_6_) *δ* 159.70, 155.32, 145.96, 142.70, 142.06, 130.65, 129.65, 127.49, 125.43, 117.35, 105.15, 99.29, 55.76, 55.52. MS *m*/*z*: 508.

#### *N* ^1^,*N*^3^-Di(naphthalen-1-yl)benzene-1,3-disulfonamide

Mp 276–277 °C, ^1^H NMR (250 MHz, DMSO-*d*_6_) *δ* 10.55 (s, 1H), 8.92 (d, *J* = 5.6 Hz, 1H), 8.12 (d, *J* = 9.8 Hz, 2H), 7.99 (d, *J* = 8.4 Hz, 3H), 7.84 (dt, *J* = 23.4, 8.7 Hz, 6H), 7.73–7.17 (m, 11H), 6.97 (d, *J* = 7.4 Hz, 1H). ^13^C NMR (63 MHz, DMSO-*d*_6_) *δ* 146.46, 142.31, 141.56, 134.36, 134.16, 132.24, 131.15, 130.91, 130.10, 129.65, 128.76, 128.43, 127.92, 127.64, 127.40, 127.23, 126.86, 126.75, 126.63, 126.02, 125.85, 125.45, 124.05, 123.55, 122.62, 120.35. MS *m*/*z*: 488.

#### *N* ^1^,*N*^3^-Bis(3-bromophenyl)benzene-1,3-disulfonamide

Mp 240–242 °C, ^1^H NMR (250 MHz, DMSO-*d*_6_) *δ* 10.79 (s, 1H), 8.19 (s, 1H), 7.97 (d, *J* = 8.2 Hz, 1H), 7.75 (t, *J* = 7.9 Hz, 1H), 7.17 (d, *J* = 17.1 Hz, 3H), 7.01 (d, *J* = 8.0 Hz, 1H). ^13^C NMR (63 MHz, DMSO-*d*_6_) *δ* 147.34, 140.72, 139.04, 132.34, 131.63, 131.43, 131.12, 128.35, 127.78, 125.37, 123.45, 123.11, 122.35, 119.24, 118.68, 116.68, 113.46. MS *m*/*z*: 546.

#### *N* ^1^,*N*^3^-Bis(4-bromophenyl)benzene-1,3-disulfonamide

Mp 330–332 °C; ^1^H NMR (250 MHz, DMSO-*d*_6_) *δ* 10.67 (s, 2H), 8.14 (s, 1H), 7.88 (s, 3H), 7.54–7.10 (m, 4H), 6.96 (s, 4H). ^13^C NMR (63 MHz, DMSO-*d*_6_) *δ* 146.33, 140.63, 136.80, 132.57, 131.94, 131.40, 128.25, 125.47, 122.72, 117.47, 117.31. MS *m*/*z*: 546.

#### *N* ^1^,*N*^3^-Bis(2,3-dimethylphenyl)benzene-1,3-disulfonamide

Mp 298–300 °C;^1^H NMR (250 MHz, DMSO-*d*_6_) *δ* 9.84 (s, 1H), 8.00 (s, 1H), 7.83 (s, 1H), 7.71 (t, *J* = 7.6 Hz, 1H), 7.00 (s, 1H), 6.93 (d, *J* = 7.1 Hz, 1H), 6.59 (s, 1H), 2.14 (s, 3H), 1.92 (s, 3H). ^13^C NMR (63 MHz, DMSO-*d*_6_) *δ* 142.07, 138.77, 138.27, 134.45, 131.69, 130.95, 130.85, 129.42, 128.93, 128.08, 126.70, 125.95, 125.33, 125.22, 121.03, 20.52, 20.23, 14.55, 13.85. MS *m*/*z*: 444.

#### *N* ^1^,*N*^3^-Di-*o*-tolylbenzene-1,3-disulfonamide

Mp 300–302 °C, ^1^H NMR (250 MHz, DMSO-*d*_6_) *δ* 9.90 (s, 2H), 8.2–7.62 (m, 4H), 6.99 (d, *J* = 59.4 Hz, 8H), 1.97 (s, 5H). ^13^C NMR (63 MHz, DMSO-*d*_6_) *δ* 145.39, 142.21, 134.94, 134.65, 132.22, 131.34, 130.99, 128.07, 127.29, 127.07, 126.87, 126.33, 125.09, 122.25,18.08. MS *m*/*z*: 416.

#### *N* ^1^,*N*^3^-Bis(4-iodophenyl)benzene-1,3-disulfonamide

Mp 302–305 °C, ^1^H NMR (250 MHz, DMSO-*d*_6_) *δ* 10.67 (s, 1H), 8.20 (s, 1H), 7.92 (d, *J* = 7.4 Hz, 1H), 7.72 (d, *J* = 7.4 Hz, 1H), 7.53 (d, *J* = 8.3 Hz, 2H), 6.82 (d, *J* = 8.3 Hz, 2H). ^13^C NMR (63 MHz, DMSO-*d*_6_) *δ* 144.76, 143.87, 143.64, 140.66, 138.41, 137.98, 137.31, 131.48, 131.40, 126.67, 125.51, 122.82, 119.71, 89.38.

#### *N* ^1^,*N*^3^-Di(pyridin-2-yl)benzene-1,3-disulfonamide

Mp 290–292 °C; ^1^H NMR (250 MHz, DMSO-*d*_6_) *δ* 13.59 (s, 2H), 8.92 (s, 5H), 8.56 (s, 3H), 8.08 (t, *J* = 49.6 Hz, 6H), 7.70 (s, 1H), 7.15 (d, *J* = 8.3 Hz, 2H), 6.81 (s, 1H). ^13^C NMR (63 MHz, DMSO-*d*_6_) *δ* 154.20, 146.78, 144.33, 143.82, 142.27, 142.01, 141.21, 135.90, 130.45, 129.77, 129.17, 127.78, 127.04, 124.56, 123.93, 115.05, 114.76, 113.89, 112.47. MS *m*/*z*: 390.

#### *N* ^1^,*N*^3^-Bis(4-fluorophenyl)benzene-1,3-disulfonamide

Mp 326–328 °C ^1^H NMR (250 MHz, DMSO-*d*_6_) *δ* 10.43 (s, 2H), 8.08 (s, 2H), 7.99–7.72 (m, 2H), 7.70 (s, 2H), 7.00 (s, 8H). ^13^C NMR (63 MHz, DMSO-*d*_6_) *δ* 159.85 (d, *J* = 242), 140.62, 133.48, 131.20 (d, *J* = 7.0), 128.17, 125.47, 123.90 (d, *J* = 8.19 Hz), 116.35 (d, *J* = 22.68 Hz), MS *m*/*z*: 424.

#### *N* ^1^,*N*^3^-Bis(2,6-dimethylphenyl)benzene-1,3-disulfonamide

Mp 307–308 °C, ^13^C NMR (63 MHz, DMSO-d_6_) *δ* 142.18, 136.67, 135.01, 131.88, 130.93, 128.02, 127.35, 125.05, 40.59, 40.26, 39.92, 39.59, 39.26, 20.84, 17.96. MS *m*/*z*: 444.

## 3. Results and discussions

**Fig. 1:**
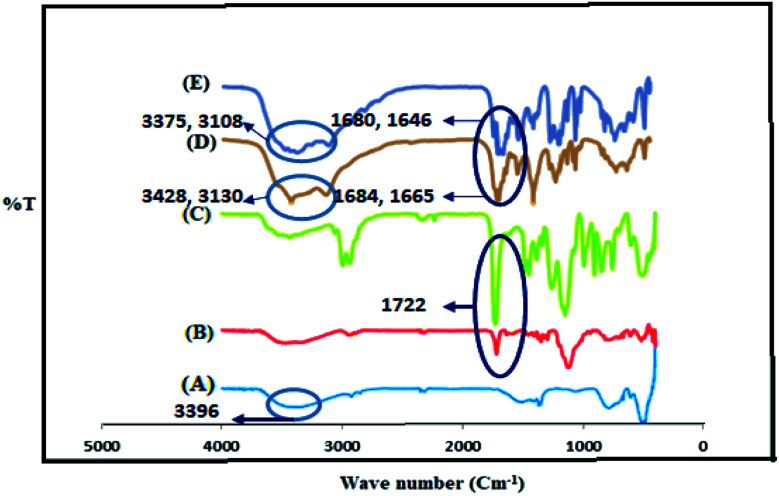
FT-IR spectra of LDH (A), LDH@MPS (B), LDH@MPS-GMA (C), LDH@MPS-GMA-TZ (D) and LDH@MPS-GMA-TZ-CuI (E).

The synthesized LDH@MPS@GMA-TZ-CuI catalyst was fully characterized by FT-IR, TGA, ICP-MS, and FESEM-EDX mapping techniques. The FT-IR spectrum of Cu–Zn–Al LDH, LDH@MPS, LDH@MPS-GMA, LDH@MPS-GMA-TZ and LDH@MPS-GMA-TZ-CuI is displayed in Fig. 1. The results obtained from FT-IR spectrum show that: (I) the presence of LDHs coated by silica (LDH@MPS), (II) the presence of glycidyl methacrylate linker (LDH@MPS-GMA), (III) the presence of thiazole moieties (IV), and the interaction of CuI NPs with the prepared support. FT-IR spectrum of Cu–Zn–Al LDH displayed vibration bands at 500 and 786 cm^−1^ (due to stretching of M–O and O–M–O bonds). The broad peaks at 3396 cm^−1^ (due to hydroxy group) can also be seen in the LDH structure (Fig. 1A). The FT-IR spectrum of LDH@MPS displayed new bands at 1722 cm^−1^ are related to ester carbonyl groups.^17^ The presence of this peak confirmed successful grafting of MPS on the surface of LDH. In Fig. 1C, the presence of the new sharp band at 1722 cm^−1^ (due to the ester carbonyl groups) was also confirmed the successful polymerization of glycidyl methacrylate.^18^ After grafting of 2-aminothiazole, the next spectrum indicated the new peak at 1646 cm^−1^ corresponds to C=N group (Fig. 1D). In addition, the ester carbonyl peaks shifted from 1722 cm^−1^ to 1684 cm^−1^. In case of LDH@MPS-GMA-TZ-CuI (Fig. 1 E), after interaction of CuI NPs with prepared support, the band at 3392 cm^−1^ (due to the NH_2_ stretching) shifted to lower wave number (3428 and 3130 to 3375 and 3108 cm^−1^). In addition, the C=N peaks shifted from 1665 to 1646 cm^−1^ (Fig. 1E).

**Fig. 2:**
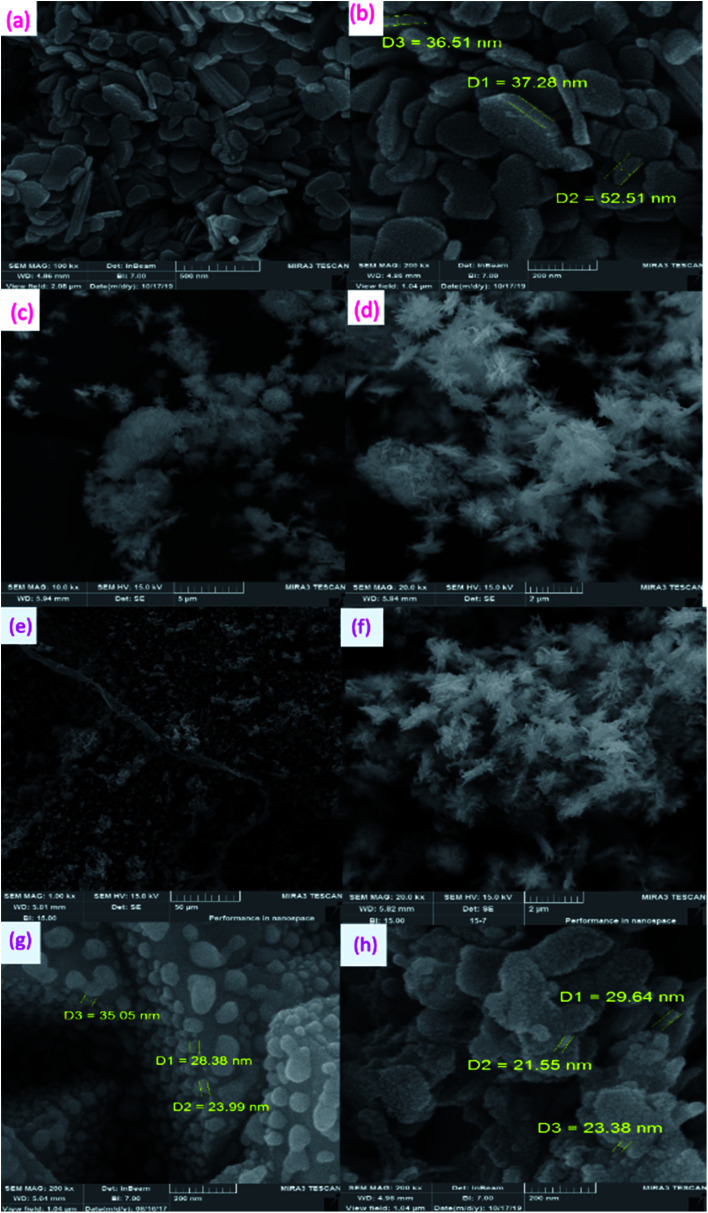
SEM image of (a and b) Cu–Zn–Al LDHs, (c and d) LDH@MPS, (e and f) LDH@MPS-GMA-TZ, (g) CuI NPs, and (h) LDH@MPS-GMA-TZ-CuI.

The FE-SEM images of Cu–Zn–Al LDH (Fig. 2a and b), LDH@MPS (Fig. 2c and d), LDH@MPS-GMA-TZ (Fig. 2e and f), CuI NPs (Fig. 2g) and the final synthesized catalyst (LDH@MPS-GMA-TZ-CuI) (Fig. 2h) are shown in Fig. 2. The FE-SEM images of LDHs indicated that Cu–Zn–Al LDH was grown in the form of sheet (Fig. 2a and b). FE-SEM image of LDH@MPS confirmed the attachment of 3-(trimethoxysilyl) propyl methacrylate to the LDHs surface (Fig. 2c and d). FE-SEM image of LDH@MPS-GMA-TZ clearly indicated the successful grafting of organic groups on the surface of LDHs (Fig. 2e and f). The FE-SEM micrograph of CuI NPs showed spherical CuI NPs were successfully fabricated in nanometric size (Fig. 2g). FE-SEM image of the LDH@MPS-GMA-TZ-CuI (Fig. 2h) showed that the CuI nanoparticles were successfully immobilized on the surface of LDHs@MPS-GMA-TZ support.

**Fig. 3:**
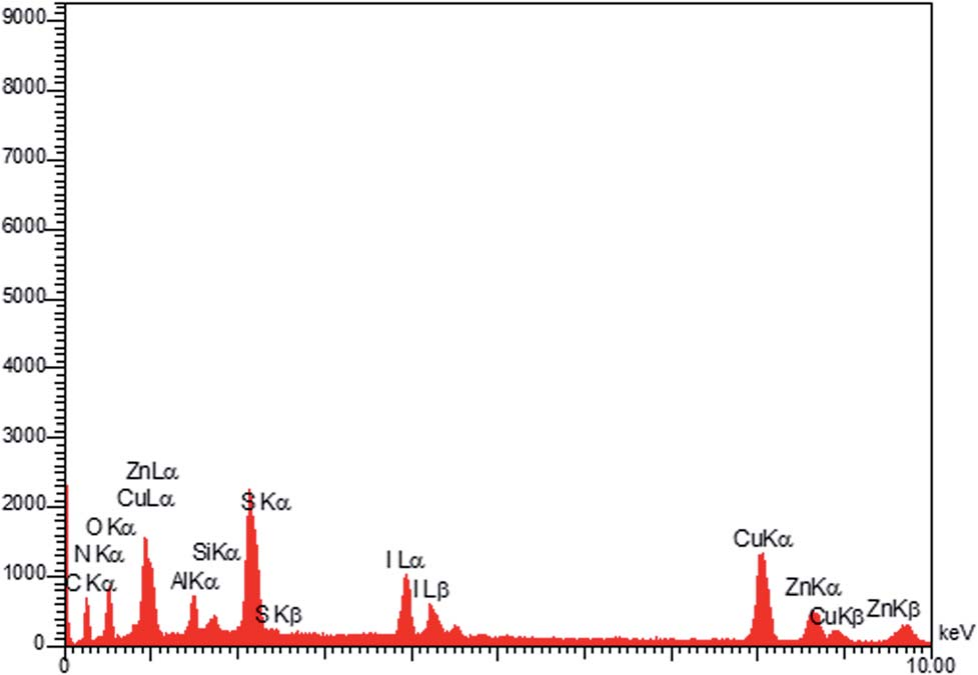
EDX analysis of LDH@MPS-GMA-TZ-CuI.

**Fig. 4:**
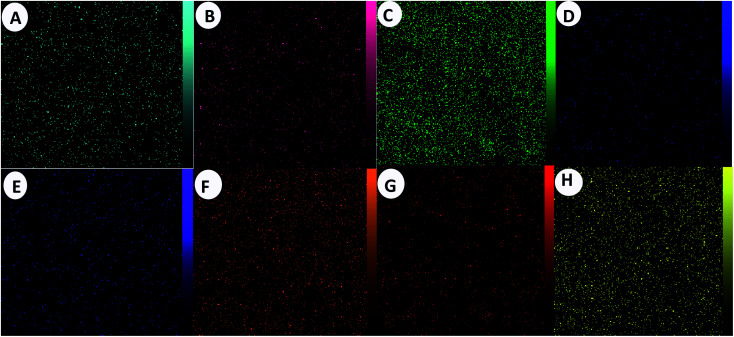
Elemental mapping of the A (Zn), B (S), C (O), D (N), E (I), F (Cu), G (C), H (Al) atoms achieved from SEM micrographs.

The EDX technique was applied for studying the elemental analysis of the prepared final catalyst (Fig. 3). The presence of all elements (C, N, Cu, Zn, S, Al, I and O) in the catalyst was proved by this technique. Elemental mapping exhibited the uniform distribution of all the elements, as shown in Fig. 4.

**Fig. 5:**
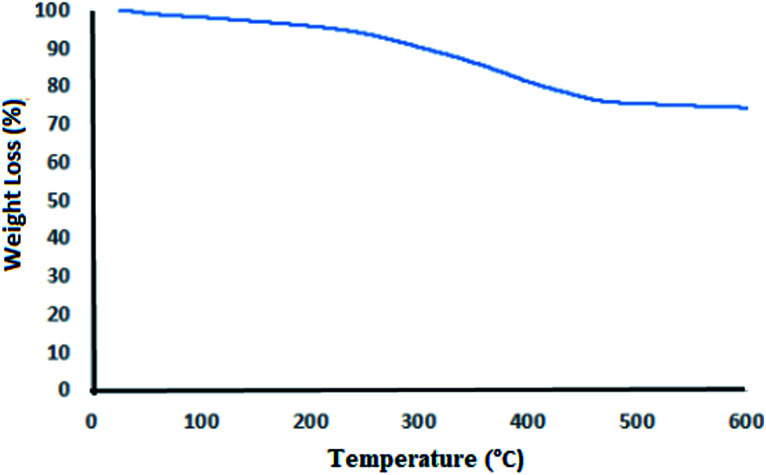
Thermogravimetric diagram of LDH@MPS-GMA-TZ-CuI nanocomposite.

Fig. 5 shows the TGA curve of LDH@MPS-GMA-TZ-CuI in which a small loss weight from 30 to 100 °C was witnessed, clearly connected to the physically absorbed water. After this, the weight decrease shown in the range of 220–450 °C. This weight loss clearly indicated the degradation of organic ligand and linker that immobilized on the surface of LDHs.

**Fig. 6:**
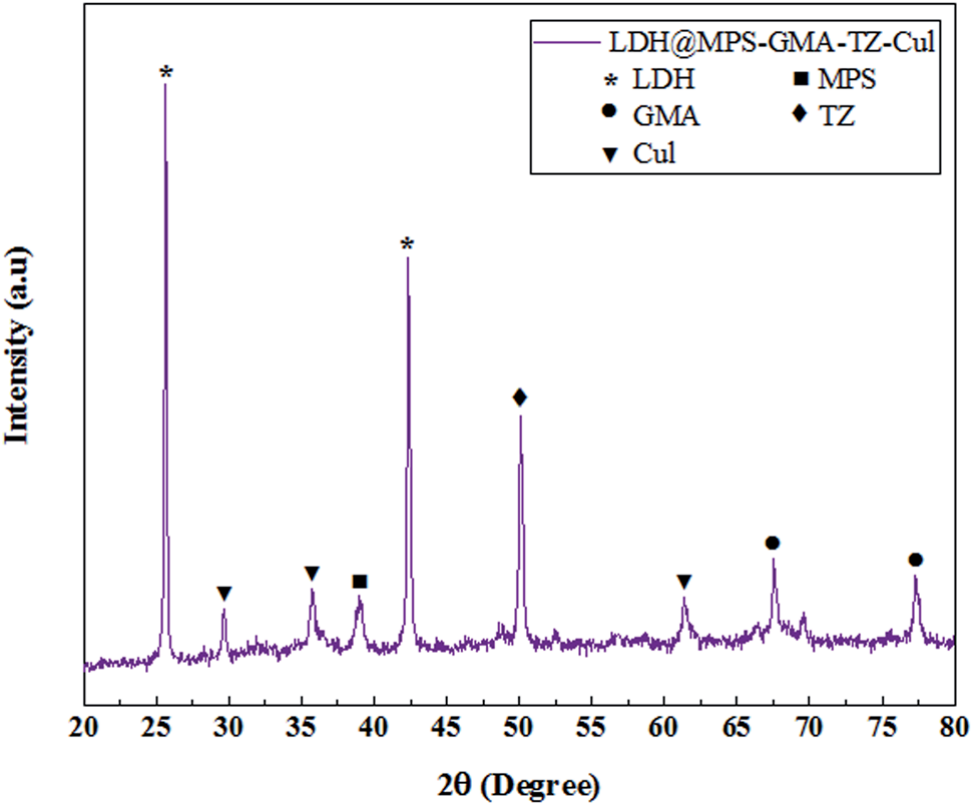
XRD pattern of LDH@MPS-GMA-TZ-CuI nanocomposite.

The crystallinity and phases structure of the LDH@MPS-GMA-TZ-CuI nanocomposite was ascertained by XRD and presented in Fig. 6. The sharp peaks at 2*θ* = 25.64°, 42.36° was attributed to the Cu–Zn–Al LDHs,^19^ the diffraction peaks appeared at 2*θ* = 29°, 35° and 61.30° can be attributed to copper iodide nanoparticles.^20^ Additionally, the diffraction peaks at 2*θ* = 67.52° and 77.32° (due to the presence of GMA)^21^ and the diffraction peaks at 2*θ* = 38.95° (due to the presence of MPS)^22^ confirms the successful coating and polymerization of LDH material. The sharp peak at 2*θ* = 50.12° is a strong reason for the presence of thiazole groups. The location and intensity of the peaks indicate the correct synthesis and high crystallinity of the prepared composite. Using the Scherer equation, the size of the crystals is calculated to be 14.1 nm. Also, the absence of additional impurities related to impurities indicates the efficiency of the proposed method in synthesis.

**Fig. 7:**
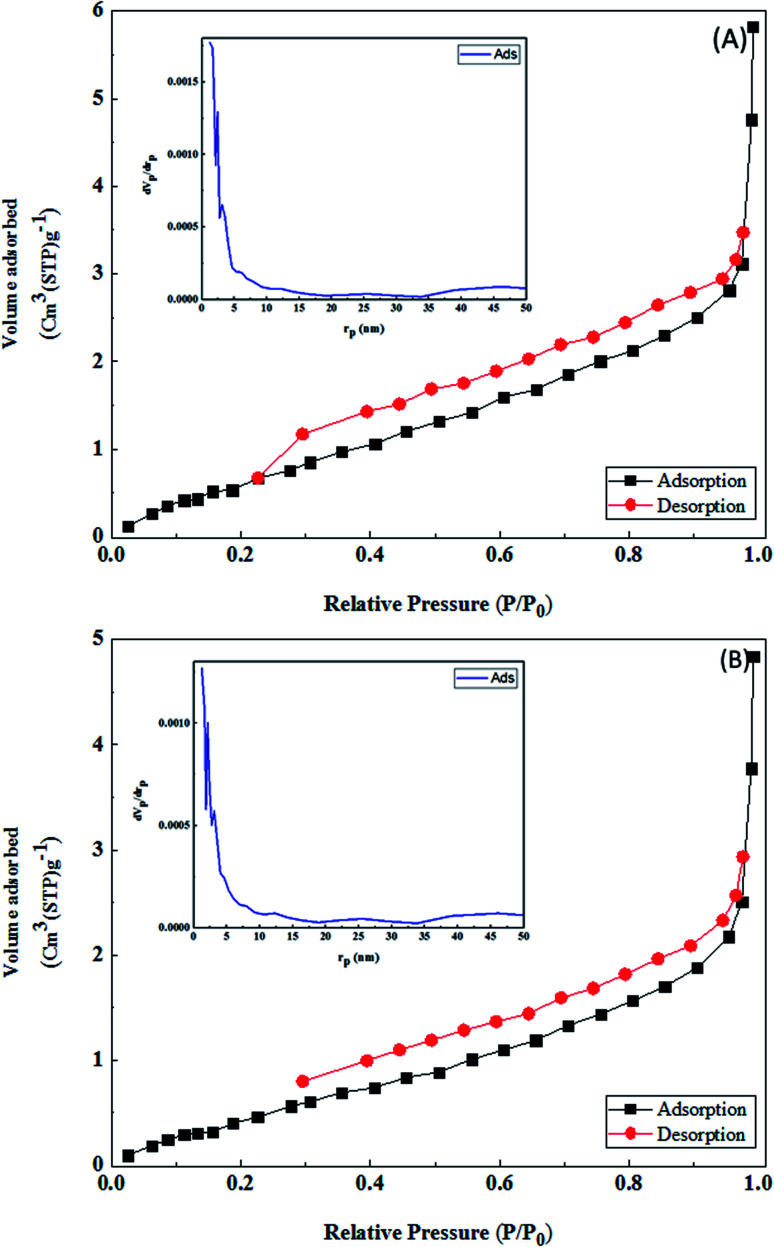
The N_2_ adsorption–desorption isotherm and BJH pore size distribution for the (A) LDH@MPS-GMA-TZ, (B) LDH@MPS-GMA-TZ-CuI.

The N_2_ adsorption–desorption isotherms of the LDH@MPS-GMA-TZ (Fig. 7A) and LDH@MPS-GMA-TZ-CuI (Fig. 7B) were measured in order to determine the textural properties. It can be seen from Fig. 7 that both LDH@MPS-GMA-TZ and LDH@MPS-GMA-TZ-CuI indicate a typical type IV isotherm with type H3 hysteresis (defined by IUPAC),^23^ which are identified as mesoporous materials. The BJH method is used to obtain the pore size distribution. As can be seen, although the maximum frequency of pores with a radius of 2.31 nm is micro pore and mesopore , and there is a heterogeneous distribution of pores in the structure. The average pore size is 1.21 nm, which is in the mesopore range. The changes associated to the textural properties of the final catalyst (LDH@MPS-GMA-TZ-CuI) can be due to the fact that copper nanoparticles which were distributed inside the LDH@MPS-GMA-TZ cavities (Table 1).

**Table 1:** Results of the Langmuir and BET measurements

Parameter	LDH@MPS-GMA-TZ-CuI	LDH@MPS-GMA-TZ
*a*_s_ (m^2^ g^−1^)	86.614	153.51
*V*_m_ (cm^3^(STP) g^−1^)	19.9	35.27
*V*_p_ (cm^3^ g^−1^)	0.0072	0.0087
*a*_p_ (m^2^ g^−1^)	3.11	4.30

After the fully characterization of prepared LDH@MPS-GMA-TZ-CuI catalyst, we aimed at analyzing the optimization of reaction parameters, *i.e.* temperature, catalyst loading and solvent over LDH@MPS-GMA-TZ-CuI for the model reaction (1,3-disulfonyl chloride and nitrobenzene). The results are presented in Table 2. As per catalytic results in different solvents (entries 1–8), a mixture of dichloroethane (DCE) with water (1 : 1 in volume) gave the best results (entry 8), and changing the ratio of solvents led to a decrease in the efficiency of the reaction. As the amount of catalyst increased (entries 8–11), the yield of the product increased rapidly in 0.05 g of the prepared catalyst (entry 8). By investigation of the effect of bases on the reaction progress, the reaction reached 92% yield after 0.5 h with pyridine at room temperature. Other inorganic bases also showed good yields (entries 13–17). However, a low yield was obtained in the absence of base (entry 18). In optimization of the temperature, increasing the temperature (up to 50 °C) negatively affected the progress of the model reaction (entry 19). The model reaction was then performed in the different amounts of sodium borohydride (0.5, 1, and 2 mmol). It was found that 0.5 mmol of NaBH_4_ provided the desired product with excellent yield (entries 20–21). Under this condition, the catalytic performance of the LDH@MPS@GMA-TZ-CuI was further examined and compared to LDH and different amount of CuI NPs (entries 22–26). The results showed that the grafting of organic groups and immobilization of the metal increased its catalytic activity, shortened the reaction time and improved the product efficiency.

**Table 2:** Screening of the reaction conditions for the synthesis of 3a^a^

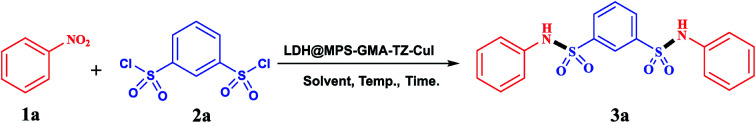						
Entry	Reductant (mmol)	Cat. (mg)	Solvent	Base	Time (h)	Yield^b^ (%)
1	NaBH_4_ (1)	50	EtOH	Pyridine	2	Trace
2	NaBH_4_ (1)	50	CH_3_CN	Pyridine	2	45
3	NaBH_4_ (1)	50	Ethyl acetate	Pyridine	2	15
4	NaBH_4_ (1)	50	H_2_O	Pyridine	2	Trace
5	NaBH_4_ (1)	50	DMF	Pyridine	2	7
6	NaBH_4_ (1)	50	CH_2_Cl_2_	Pyridine	2	55
7	NaBH_4_ (1)	50	C_2_H_4_Cl_2_	Pyridine	2	78
8	NaBH_4_ (1)	50	H_2_O : C_2_H_4_Cl_2_	Pyridine	0.5	92
9	NaBH_4_ (1)	40	H_2_O : C_2_H_4_Cl_2_	Pyridine	0.5	88
10	NaBH_4_ (1)	20	H_2_O : C_2_H_4_Cl_2_	Pyridine	1	60
11	NaBH_4_ (1)	10	H_2_O : C_2_H_4_Cl_2_	Pyridine	1	40
12	NaBH_4_ (1)	60	H_2_O : C_2_H_4_Cl_2_	Pyridine	0.5	92
13	NaBH_4_ (1)	50	H_2_O : C_2_H_4_Cl_2_	Et_3_N	2	41
14	NaBH_4_ (1)	50	H_2_O : C_2_H_4_Cl_2_	K_2_CO_3_	2	56
15	NaBH_4_ (1)	50	H_2_O : C_2_H_4_Cl_2_	Na_2_CO_3_	2	50
16	NaBH_4_ (1)	50	H_2_O : C_2_H_4_Cl_2_	KOH	2	70
17	NaBH_4_ (1)	50	H_2_O : C_2_H_4_Cl_2_	NaHCO_3_	2	60
18	NaBH_4_ (1)	50	H_2_O : C_2_H_4_Cl_2_	—	24	40
19	NaBH_4_ (1)	50	H_2_O : C_2_H_4_Cl_2_	Pyridine	0.5	88^c^
20	NaBH_4_ (2)	50	H_2_O : C_2_H_4_Cl_2_	Pyridine	0.5	92
21	NaBH_4_ (0.5)	50	H_2_O : C_2_H_4_Cl_2_	Pyridine	2	60
22	NaBH_4_ (1)	50	H_2_O : C_2_H_4_Cl_2_	Pyridine	12	Trace^d^
23	NaBH_4_ (1)	50	H_2_O : C_2_H_4_Cl_2_	Pyridine	12	50^e^
24	NaBH_4_ (1)	40	H_2_O : C_2_H_4_Cl_2_	Pyridine	12	39^f^
25	NaBH_4_ (1)	70	H_2_O : C_2_H_4_Cl_2_	Pyridine	12	59^g^
26	NaBH_4_ (1)	100	H_2_O : C_2_H_4_Cl_2_	Pyridine	12	72^h^

a Reaction conditions: nitroarene (0.5 mmol), LDH@MPS-GMA-TZ-CuI (5 mg), 1,3-disulfonylchloride (0.25 mmol), pyridine (0.5 mmol), NaBH_4_ (1.0 mmol) and H_2_O : C_2_H_4_Cl_2_ (1 : 1, 2 mL), at room temperature.

b Isolated yield.

c The reaction was investigated at 50 °C.

d The reaction was investigated in the presence of Cu–Zn–Al LDH.

e The reaction was investigated in the presence of CuI NPs (50 mg).

f The reaction was investigated in the presence of CuI NPs (40 mg).

g The reaction was investigated in the presence of CuI NPs (70 mg).

h The reaction was investigated in the presence of CuI NPs (100 mg).

Under the optimal conditions, we evaluated its catalytic efficiency for synthesis of bis-*N*-aryl sulfonamides from various substituted nitroarenes (Table 3). We found that a variety of functionalized bis-*N*-aryl sulfonamides are accessible in good to excellent yields using this method. With regard to the scope of nitroarenes, electron-donating nitrobenzenes such as 2,4-dimethoxy, 2,4-dimethyl, 2,6-dimethyl, 2,3-dimethyl, *o*-CH_3_ (3b–h), electron-withdrawing substituted nitrobenzenes such as *p*-Br*, p*-Cl*, p*-I*, p*-F*, m*-NO_2_, (3i–m), heterocyl nitroarene like 2-nitropyridine (3n), and 1-nitronaphthalene (3o) are all good substrates. Compared with electron-withdrawing nitroarenes, nitroarenes with electron-donating substituents gave the desired products in a better yields. In addition, the reaction was performed with nitrobenzenes containing substituents at the *ortho* position (3d–g), and the efficiency of forming the desired product was good.

**Table 3:** Synthesis of bis-*N*-arylsulfonamides derivatives using LDH@MPS-GMA-TZ-CuI.^a^

Entry	Substrate	Product^b^	Time (h)	Yield^c^ (%)
1	Nitrobenzene	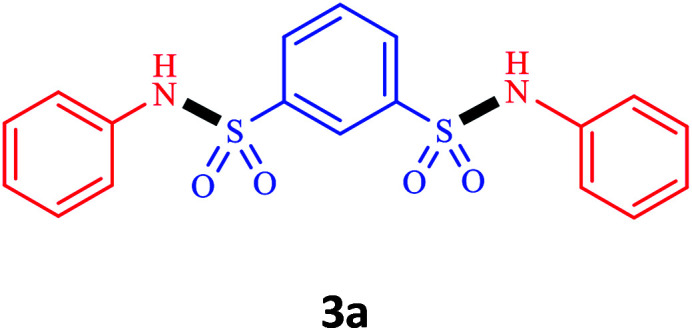	0.5	92
2	1-Methyl-4-nitrobenzene	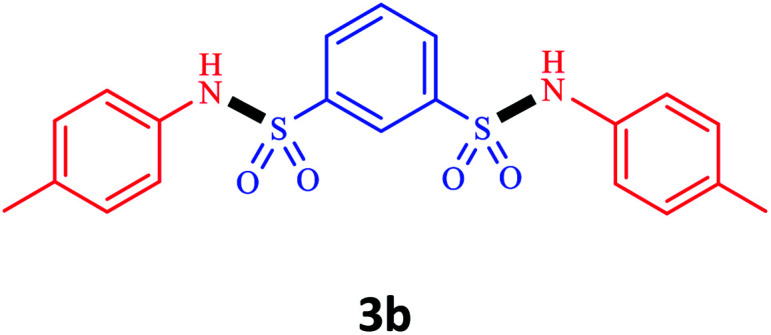	0.5	98
3	1-Methoxyl-4-nitrobenzene	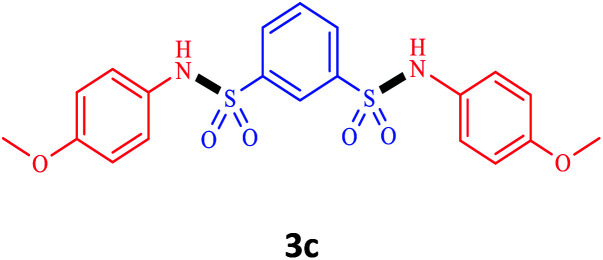	1	91
4	2,4-Dimethoxy-1-nitrobenzene	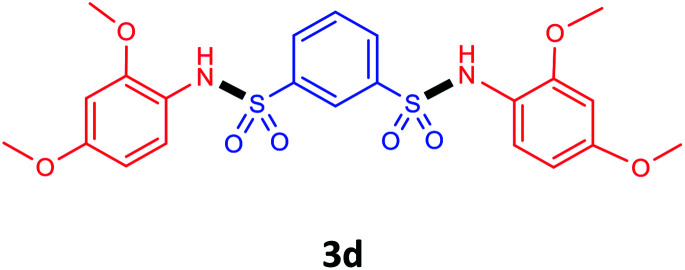	1	85
5	2,4-Dimethyl-1-nitrobenzene	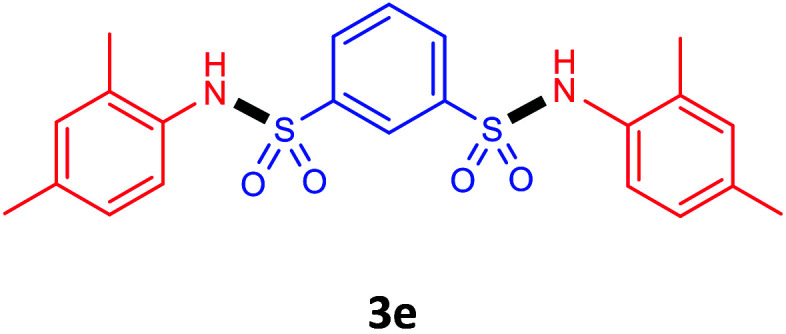	1	88
6	1,3-Dimethyl-2-nitrobenzene	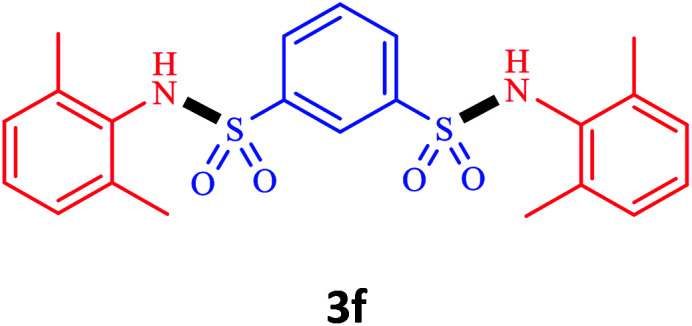	1	78
7	1,2-Dimethyl-3-nitrobenzene	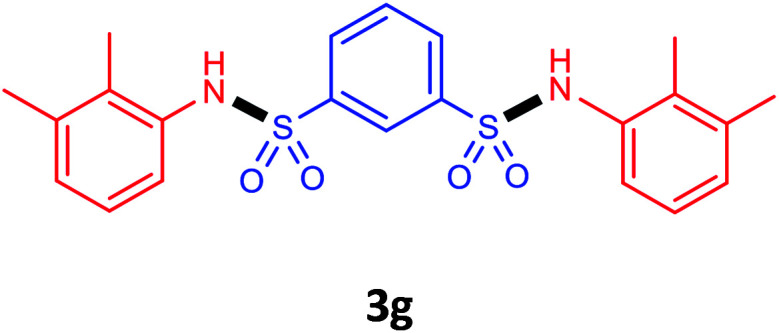	1.5	88
8	1-Methyl-2-nitrobenzene	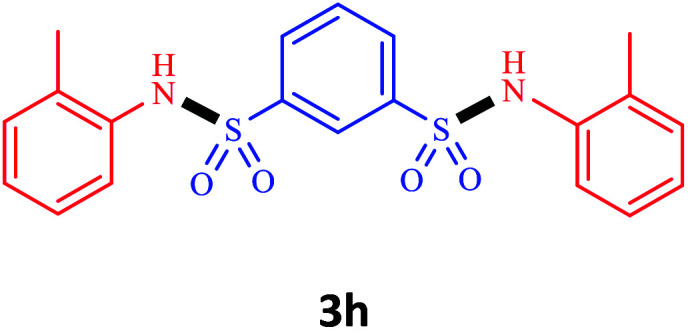	1.5	92
9	1-Bromo-4-nitrobenzene	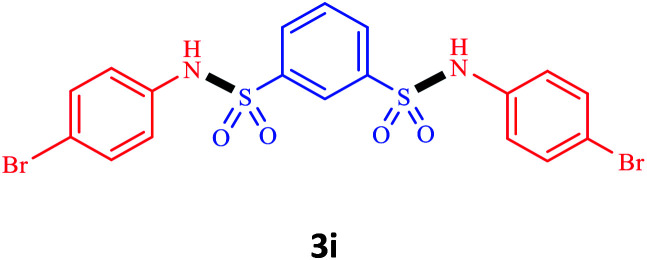	1.5	88
10	1-Chloro-4-nitrobenzene	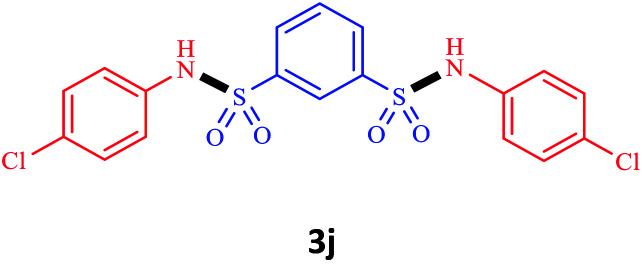	1.5	90
11	1-Iodo-4-nitrobenzene	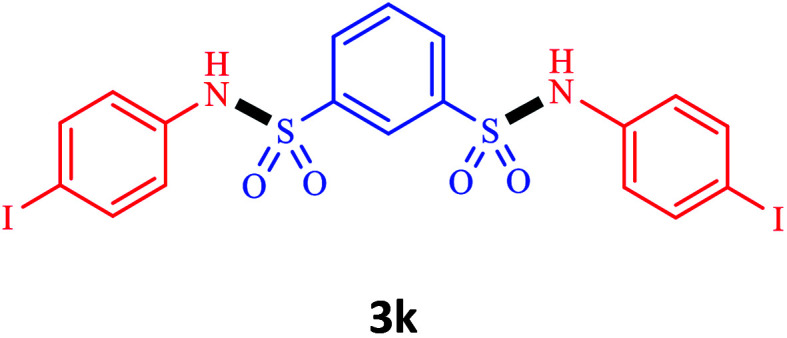	2	78
12	1-Fluoro-4-nitrobenzene	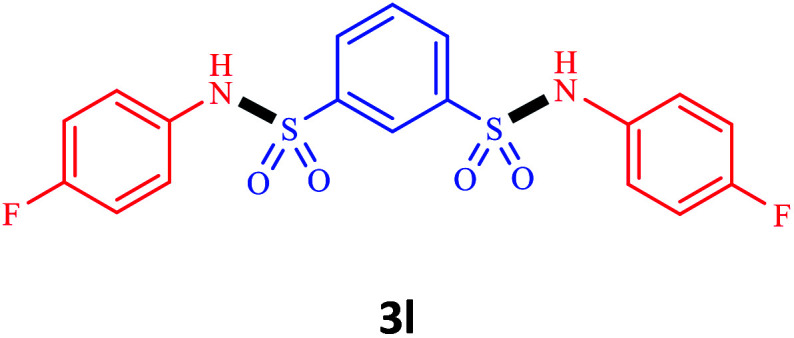	1	80
13	1-Bromo-3-nitrobenzene	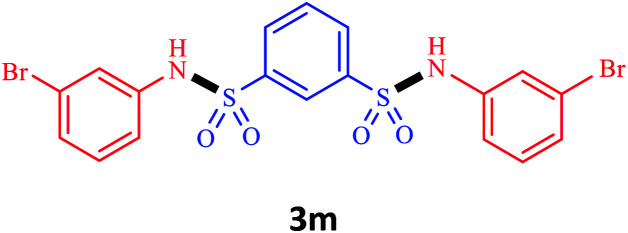	2.5	88
14	2-Nitropyridine	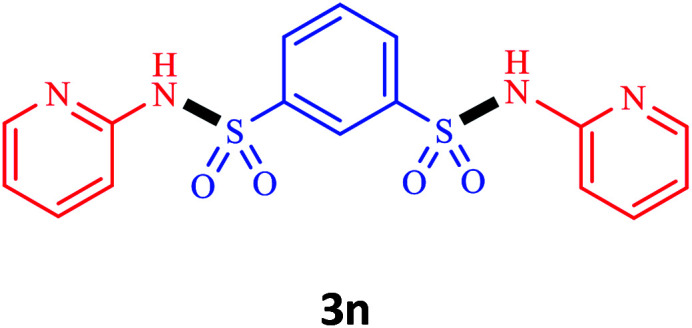	1.5	90
15	1-Nitronaphthalene	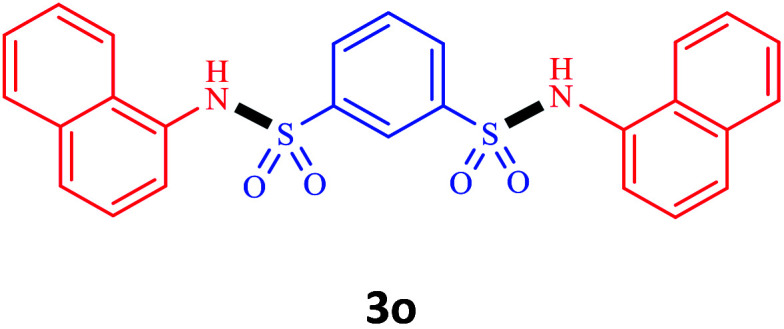	4	75

a Reaction condition: nitroarene (0.5 mmol), LDH@MPS-GMA-TZ-CuI (5 mg), 1,3-disulfonylchloride (0.25 mmol), pyridine (0.5 mmol), NaBH_4_ (1.0 mmol) and H_2_O : C_2_H_4_Cl_2_ (1 : 1, 2 mL), at room temperature.

b All the products were characterized by HNMR, CNMR, mass and FT-IR.

c Isolated yields.

### 3.1. Catalyst recycling

**Fig. 8:**
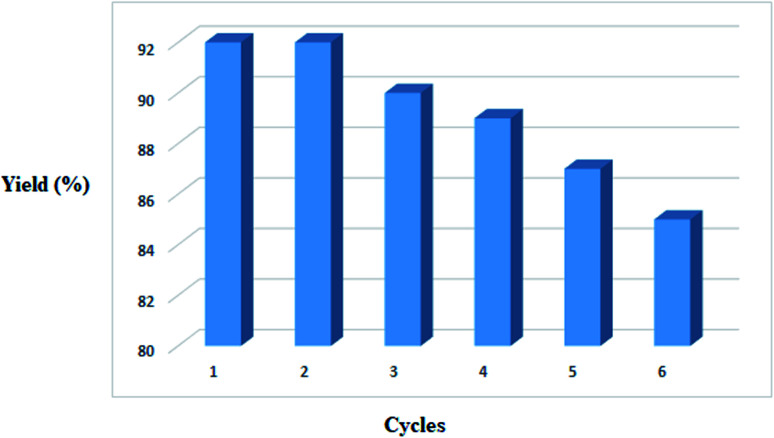
Reusability of catalyst for the model reaction (1,3-disulfonyl chloride and nitrobenzene).

Fig. 8 represents the regeneration of LDH@MPS-GMA-TZ-CuI up to six consecutive recycles. After each recycle, very little and insignificant change in the product yield has been noticed (92, 92, 90, 89, 87, 85) (Fig. 8).

The efficiency of LDH@MPS@GMA@TZ-CuI for this method was compared to some other related reports (Table 4). In the present work, using LDH@MPS-GMA-TZ-CuI as a recyclable catalyst can remarkably enhance the product yield and shorten the reaction time.

**Table 4:** Comparison of the present methodology with other reported catalysts

Entry	Conditions	Yield (%) [ref]
1	Sodium arylsulfinates (0.75 mmol), nitrobenzene (0.5 mmol), NaHSO_3_ (1.5 mmol), FeCl_2_ (10 mol%), DMDACH (20 mol%), DMSO (2 mL), 12 h, Ar	90 [6]
2	Nitrobenzene (0.25 mmol), benzene sulfonyl chlorides (0.5 mmol), Fe dust (1.0 mmol), H_2_O (1 mL), 60 °C, 36 h	85 [7]
3	Nitrobenzene (0.3 mmol), benzene sulfonyl chlorides (0.6 mmol), iron powder (1.15 mmol), water (1.5 mL), 60 °C, 40 h.	85 [8]
4	Nitrobenzene (0.5 mmol), sodium arylsulfinates (1 mmol), NaHSO_3_ (1 mmol), MIL-101(Fe) (10 mg, 8 mol% of Fe), H_2_O (2 mL), 60 °C, 20 h	91 [9]
5	A solution (80 mL) of water (phosphate buffer, pH = 3.0, *c* = 0.2 M)/ethanol mixture (20/80, v/v), arylsulfinic acids sodium salt (2 mmol), *p*-nitroaniline (1 mmol)	60 [11]
6	Nitrobenzene (0.5 mmol), LDH@MPS-GMA-TZ-CuI (5 mg), 1,3-disulfonylchloride (0.25 mmol), pyridine (0.5 mmol), NaBH_4_ (1.0 mmol) and H_2_O : C_2_H_4_Cl_2_ (1 : 1, 2 mL), room temperature, 0.5 h	92 [This work]

**Scheme 3:**
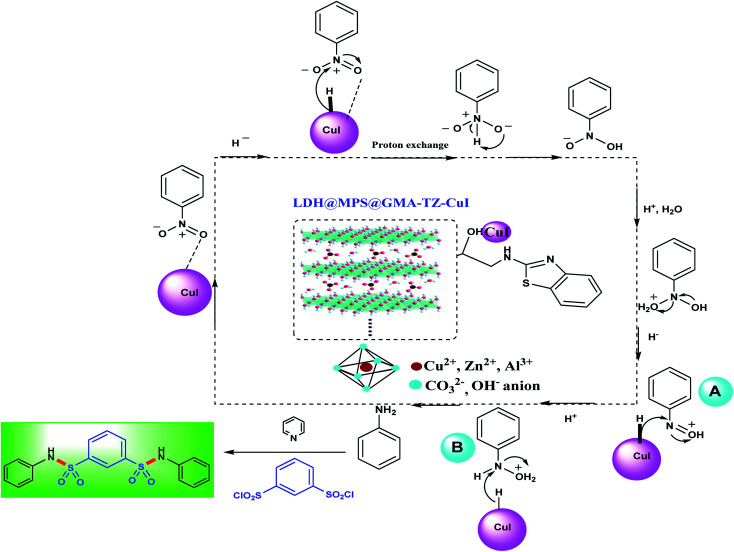
The proposed mechanism for the synthesis of bis-*N*-arylsulfonamides in the presence of LDH@MPS-GMA-TZ-CuI catalyst.

A plausible mechanism for the synthesis of bis-*N*-aryl sulfonamides from the reaction of 1,3-disulfonyl chloride and nitroarenes in the presence of LDH@MPS-GMA-TZ-CuI catalyst has been proposed in Scheme 3. According to the figure, it is believed that effective electronic interactions between copper and heteroatoms can create an appropriate substrate for this type of reaction.^24^ These close electronic interactions of heteroatoms with dissociated hydrogen atoms from sodium borohydride, onto the surface of the Cu NPs cause nitrobenzene derivatives to be adsorbed and converted to anilines during successive dehydration processes. In fact, sodium borohydride is considered as a substantial H-supporter for the reduction process and can effectively interact with Cu NPs surfaces from its nitrogen sites.

First of all, the reaction of sodium borohydride and copper can lead to forming copper hydride species. Subsequently, the hydride group transfers to nitroarene species. In mechanistic pathway, the intermediates nitrosobenzene (A) and phenyl hydroxyl amine (B) are generated, followed by water elimination and hydride transfer process to intermediate (B) and generated the aniline intermediate (Scheme 3). Finally, the bis-*N*-aryl sulfonamides were obtained from the amination of 1,3-disulfonyl chlorides in the presence of pyridine base.^25^

## 4. Conclusions

The aim of the present research was to synthesize a new type of polymer–LDH nanocomposite with thiazole for immobilization of CuI NPs. The catalytic activity of LDH@MPS-GMA-TZ-CuI was explored for effective synthesis of bis-*N*-aryl sulfonamides from the reaction of 1,3-disulfonyl chloride and nitroarenes. The results illustrated that the high metal loading results in the high performance of the catalyst for the synthesis of bis-*N*-aryl sulfonamides.

## Conflicts of interest

There are no conflicts to declare.

## Supplementary Material

RA-011-D1RA02086B-s001
