# Supplementary material for: CuI nanoparticles supported on a novel polymer-layered double hydroxide nanocomposite: an efficient heterogeneous nanocatalyst for the synthesis of bis-N-arylsulfonamides
Source: RSC Adv. 2021 May 26;11(31):19147–57. doi: 10.1039/d1ra02086b (PMC9033673; doi:10.1039/d1ra02086b)
Supplement: RA-011-D1RA02086B-s001 [file RA-011-D1RA02086B-s001.pdf]

Electronic Supplementary Material (ESI) for RSC Advances.
This journal is © The Royal Society of Chemistry 2021

Supporting Information

CuI Nanoparticles supported on novel polymer-layered double hydroxides nanocomposite an efficient heterogeneous nanocatalyst for the synthesis of *bis*-*N*-arylsulfonamides

Jamshid Babamoradi, Ramin Ghorbani-Vaghei*,Sedigheh Alavinia

Department of Organic Chemistry, Faculty of Chemistry, Bu-Ali Sina University 65174, Hamedan, Iran
Fax/Tel +98(811)8257407; E-mail: ghorbani@basu.ac.ir

Spectral data of compounds:

***N¹,N³*-di-*p*-tolylbenzene-1,3-disulfonamide:**

M.p. 282–284 °C, ^{1}H NMR (250 MHz, DMSO-d_6) δ 10.32 (s, 2H), 8.36–7.47 (m, 4H), 6.92 (d, *J* = 18.0 Hz, 8H), 2.15 (s, 6H). ^{13}C NMR (63 MHz, DMSO-d_6) δ 143.74, 138.33, 138.02, 133.41, 131.17, 130.53, 129.23, 128.91, 128.34, 127.91, 124.65, 18.99. MS m/z: 416.

***N¹,N³*-*bis*(4-methoxyphenyl)benzene-1,3-disulfonamide:**

M.p. 269–270 °C, ^{1}H NMR (250 MHz, DMSO-d_6) δ 10.12 (s, 1H), 8.07 (s, 1H), 7.81 (d, *J* = 7.8 Hz, 1H), 7.65 (t, *J* = 7.9 Hz, 1H), 6.87 (d, *J* = 8.5 Hz, 2H), 6.75 (d, *J* = 8.7 Hz, 2H), 3.72 (s, 3H). ^{13}C NMR (63 MHz, DMSO-d_6) δ 157.33, 140.86, 131.08, 130.89, 129.71, 125.50, 124.46, 114.80, 55.58. MS m/z: 448.

***N¹,N³*-*bis*(2,3-dimethoxyphenyl)benzene-1,3-disulfonamide:**

M.p. 279–281 °C; ^{1}H NMR (250 MHz, DMSO-d_6) δ 9.57 (s, 1H), 8.90 (d, *J* = 5.4 Hz, 1H), 8.20 – 7.83 (m, 1H), 7.70 (dd, *J* =7.8 Hz, 1H), 7.02 (d, *J* = 8.4 Hz, 1H), 6.68–6.15 (m, 2H), 3.68 (s, 4H), 3.26 (s, 3H). ^{13}C NMR (63 MHz, DMSO-d_6) δ 159.70, 155.32, 145.96, 142.70, 142.06, 130.65, 129.65, 127.49, 125.43, 117.35, 105.15, 99.29, 55.76, 55.52. MS m/z:508.

***N¹,N³*-di(naphthalen-1-yl)benzene-1,3-disulfonamide:**

M.p. 276–277 °C, ^{1}H NMR (250 MHz, DMSO-d_6) δ 10.55 (s, 1H), 8.92 (d, *J* = 5.6 Hz, 1H), 8.12 (d, *J* = 9.8 Hz, 2H), 7.99 (d, *J* = 8.4 Hz, 3H), 7.84 (dt, *J* = 23.4, 8.7 Hz, 6H), 7.73 – 7.17 (m, 11H), 6.97 (d, *J* = 7.4 Hz, 1H). ^{13}C NMR (63 MHz, DMSO-d_6) δ 146.46, 142.31, 141.56, 134.36, 134.16, 132.24, 131.15, 130.91, 130.10, 129.65, 128.76, 128.43, 127.92, 127.64, 127.40, 127.23, 126.86, 126.75, 126.63, 126.02, 125.85, 125.45, 124.05, 123.55, 122.62, 120.35. MS m/z:488.

***N^1,N^3-bis*(3-bromophenyl)benzene-1,3-disulfonamide:**

M.p. 240–242 °C, ^{1}H NMR (250 MHz, DMSO-d_6) δ 10.79 (s, 1H), 8.19 (s, 1H), 7.97 (d, *J* = 8.2 Hz, 1H), 7.75 (t, *J* = 7.9 Hz, 1H), 7.17 (d, *J* = 17.1 Hz, 3H), 7.01 (d, *J* = 8.0 Hz, 1H). ^{13}C NMR (63 MHz, DMSO-d_6) δ 147.34, 140.72, 139.04, 132.34, 131.63, 131.43, 131.12, 128.35, 127.78, 125.37, 123.45, 123.11, 122.35, 119.24, 118.68, 116.68, 113.46. MS m/z: 546.

***N^1,N^3-bis*(4-bromophenyl)benzene-1,3-disulfonamide:**

M.p. 330–332 °C; ^{1}H NMR (250 MHz, DMSO-d_6) δ 10.67 (s, 2H), 8.14 (s, 1H), 7.88 (s, 3H), 7.54 – 7.10 (m, 4H), 6.96 (s, 4H). ^{13}C NMR (63 MHz, DMSO-d_6) δ 146.33, 140.63, 136.80, 132.57, 131.94, 131.40, 128.25, 125.47, 122.72, 117.47, 117.31. MS m/z: 546.

***N^1,N^3-bis*(2,3-dimethylphenyl)benzene-1,3-disulfonamide:**

M.p. 298–300 °C;^{1}H NMR (250 MHz, DMSO-d_6) δ 9.84 (s, 1H), 8.00 (s, 1H), 7.83 (s, 1H), 7.71 (t, *J* = 7.6 Hz, 1H), 7.00 (s, 1H), 6.93 (d, *J* = 7.1 Hz, 1H), 6.59 (s, 1H), 2.14 (s, 3H), 1.92 (s, 3H). ^{13}C NMR (63 MHz, DMSO-d_6) δ 142.07, 138.77, 138.27, 134.45, 131.69, 130.95, 130.85, 129.42, 128.93, 128.08, 126.70, 125.95, 125.33, 125.22, 121.03, 20.52, 20.23, 14.55, 13.85. MS m/z:444.

N^1,N^3-di-*o*-tolylbenzene-1,3-disulfonamide:

M.p. 300–302 °C, ^{1}H NMR (250 MHz, DMSO-d_6) δ 9.90 (s, 2H), 8.2 –7.62 (m, 4H), 6.99 (d, *J* = 59.4 Hz, 8H), 1.97 (s, 5H). ^{13}C NMR (63 MHz, DMSO-d_6) δ 145.39, 142.21, 134.94, 134.65, 132.22, 131.34, 130.99, 128.07, 127.29, 127.07, 126.87, 126.33, 125.09, 122.25,18.08. MS m/z:416.

***N^1,N^3-bis*(4-iodophenyl)benzene-1,3-disulfonamide:**

M.p. 302–305 °C, ^{1}H NMR (250 MHz, DMSO-d_6) δ 10.67 (s, 1H), 8.20 (s, 1H), 7.92 (d, *J* = 7.4 Hz, 1H), 7.72 (d, *J* = 7.4 Hz, 1H), 7.53 (d, *J* = 8.3 Hz, 2H), 6.82 (d, *J* = 8.3 Hz, 2H). ^{13}C NMR (63 MHz, DMSO-d_6) δ 144.76, 143.87, 143.64, 140.66, 138.41, 137.98, 137.31, 131.48, 131.40, 126.67, 125.51, 122.82, 119.71, 89.38.

N^1,N^3-di(pyridin-2-yl)benzene-1,3-disulfonamide:

M.p. 290–292 °C; ^{1}H NMR (250 MHz, DMSO-d_6) δ 13.59 (s, 2H), 8.92 (s, 5H), 8.56 (s, 3H), 8.08 (t, *J* = 49.6 Hz, 6H), 7.70 (s, 1H), 7.15 (d, *J* = 8.3 Hz, 2H), 6.81 (s, 1H). ^{13}C NMR (63 MHz, DMSO-d_6) δ 154.20, 146.78, 144.33, 143.82, 142.27, 142.01, 141.21, 135.90, 130.45, 129.77, 129.17, 127.78, 127.04, 124.56, 123.93, 115.05, 114.76, 113.89, 112.47. MS m/z:390.

***N^1,N^3-bis*(4-fluorophenyl)benzene-1,3-disulfonamide:**

M.p. 326–328 °C ^{1}H NMR (250 MHz, DMSO-d_6) δ 10.43 (s, 2H), 8.08 (s, 2H), 7.99–7.72 (m, 2H), 7.70 (s, 2H), 7.00 (s, 8H). ^{13}C NMR (63 MHz, DMSO-d_6) δ 159.85 (d, *J*=242), 140.62, 133.48, 131.20 (d, *J*=7.0), 128.17, 125.47, 123.90 (d, *J* = 8.19 Hz), 116.35 (d, *J* = 22.68 Hz), MS m/z:424.

***N^1,N^3-bis*(2,6-dimethylphenyl)benzene-1,3-disulfonamide:**

307-308 °C, ^{13}C NMR (63 MHz, DMSO-d_6) δ 142.18, 136.67, 135.01, 131.88, 130.93, 128.02, 127.35, 125.05, 40.59, 40.26, 39.92, 39.59, 39.26, 20.84, 17.96. MS m/z:444.

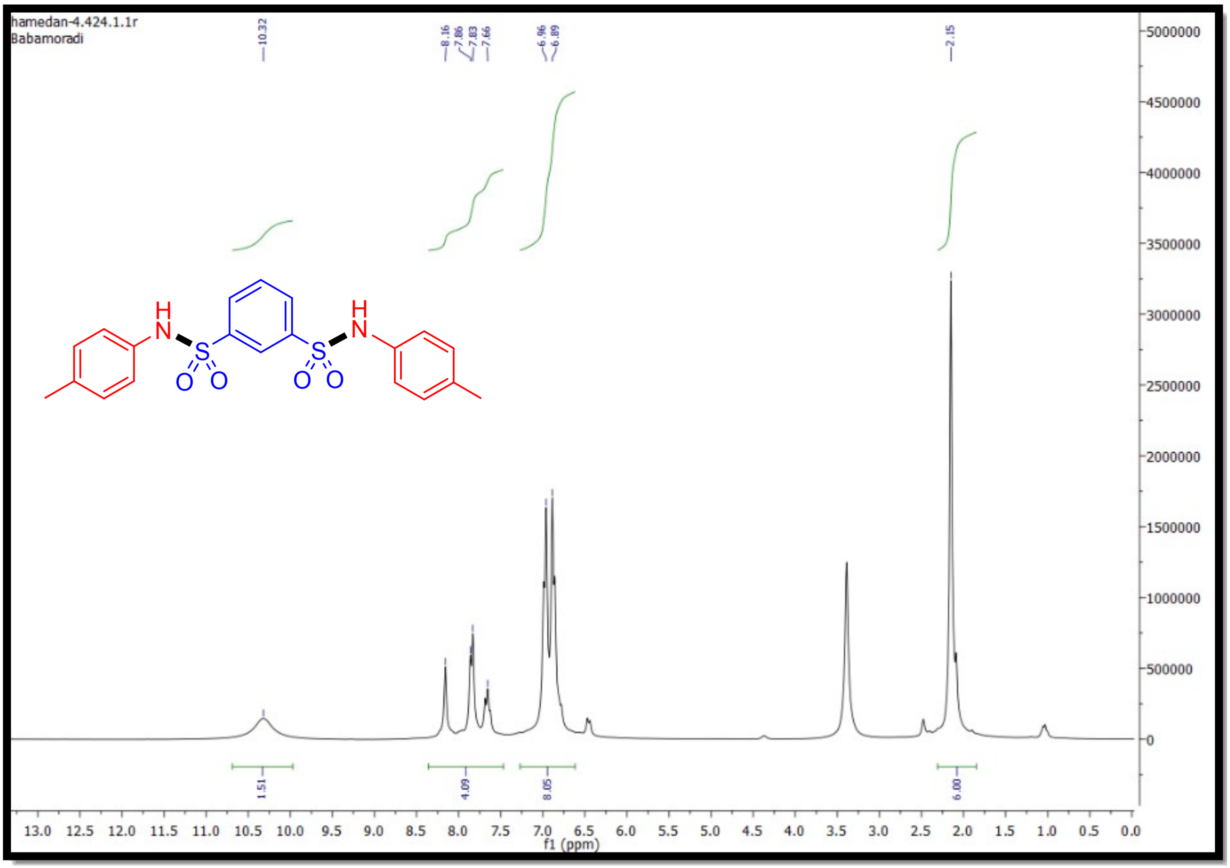
hamedan-4.424.1.1r
Babamoradi
10.32
8.16
7.86
7.83
7.66
6.96
6.89
2.15
1.51
4.09
8.05
6.00
f1 (ppm)
S
O
O
N
H
S
O
O
N
H

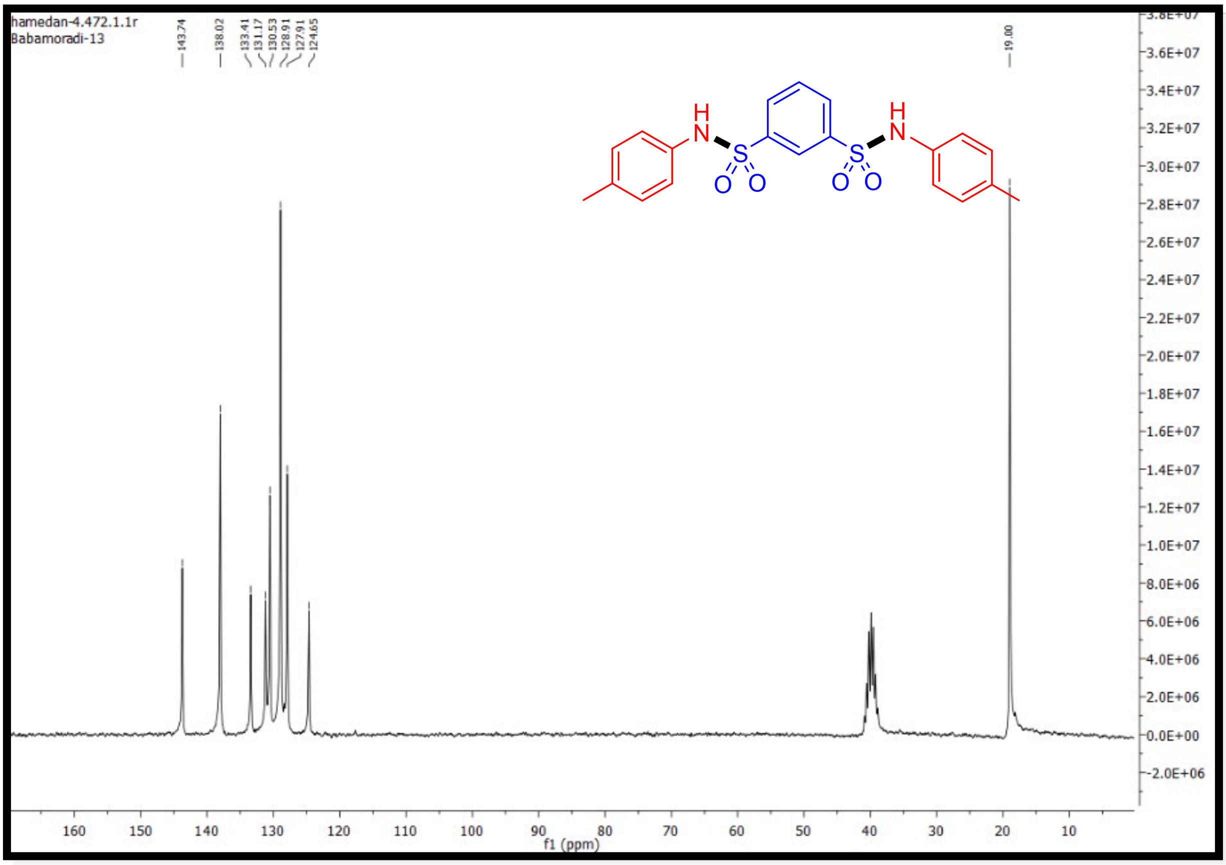
hamedan-4.472.1.1r
Babamoradi-13
143.74
138.02
133.41
131.17
130.53
128.91
127.91
124.65
19.00
H
N
S
O O
f1 (ppm)

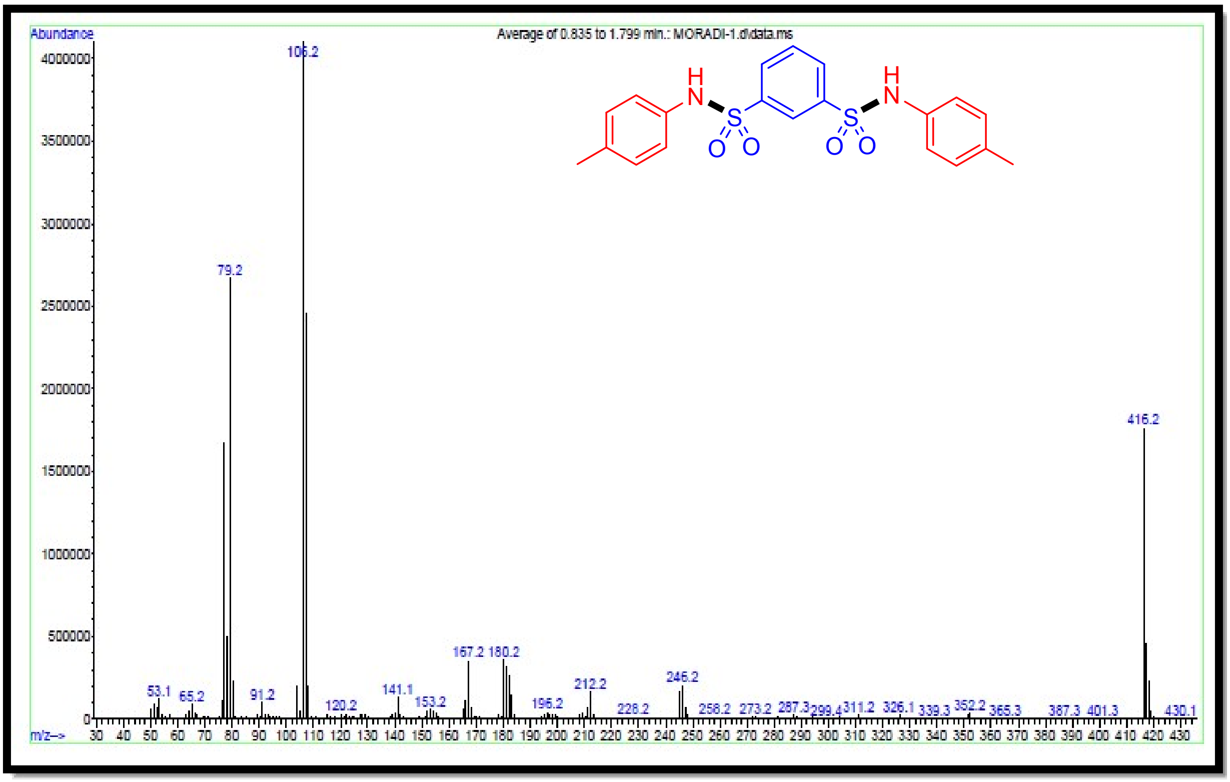
Abundance
Average of 0.835 to 1.799 min.: MORADI-1.d\data.ms
106.2
79.2
416.2
167.2
180.2
212.2
246.2
53.1
65.2
91.2
120.2
141.1
153.2
195.2
228.2
m/z-->

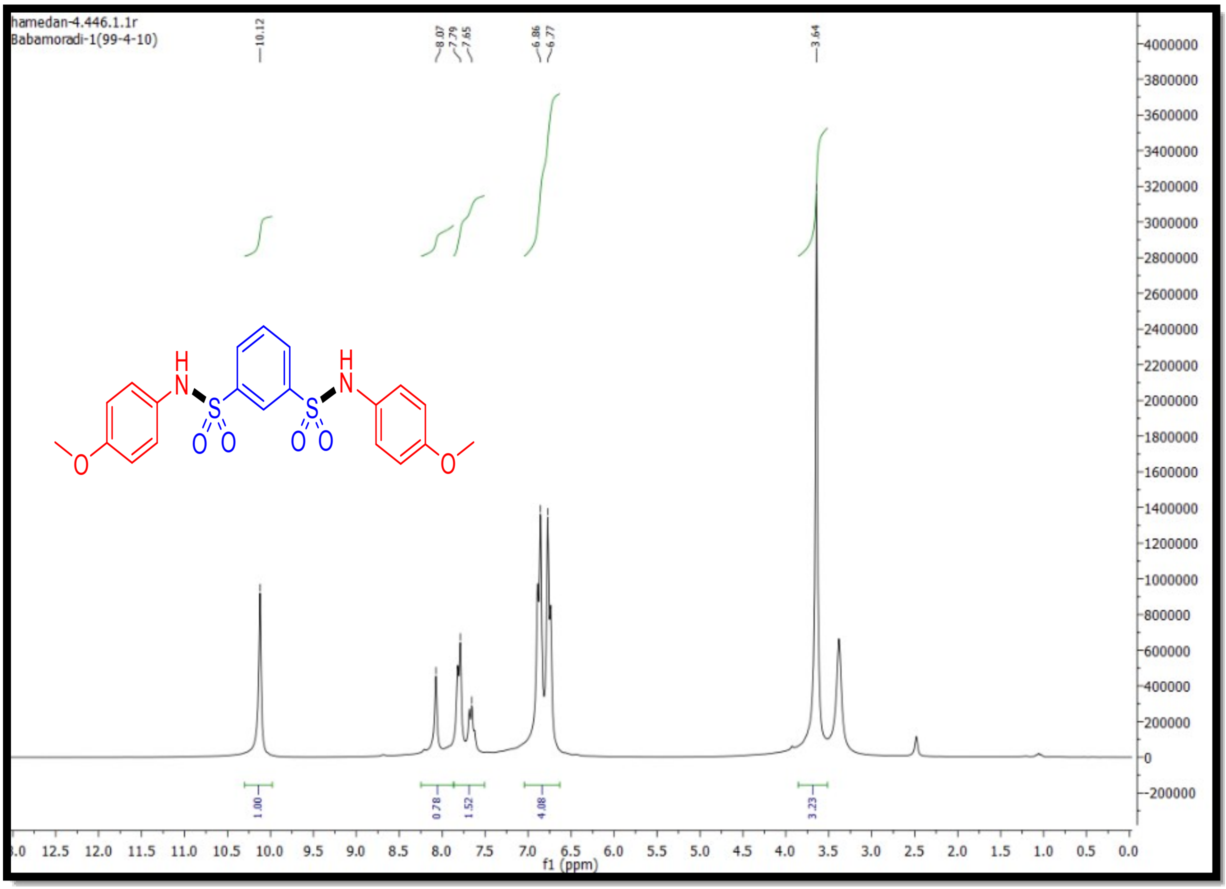
hamedan-4.446.1.1r
Babamoradi-1(99-4-10)
10.12
8.07
7.79
7.65
6.86
6.77
3.64
1.00
0.78
1.52
4.08
3.23
f1 (ppm)

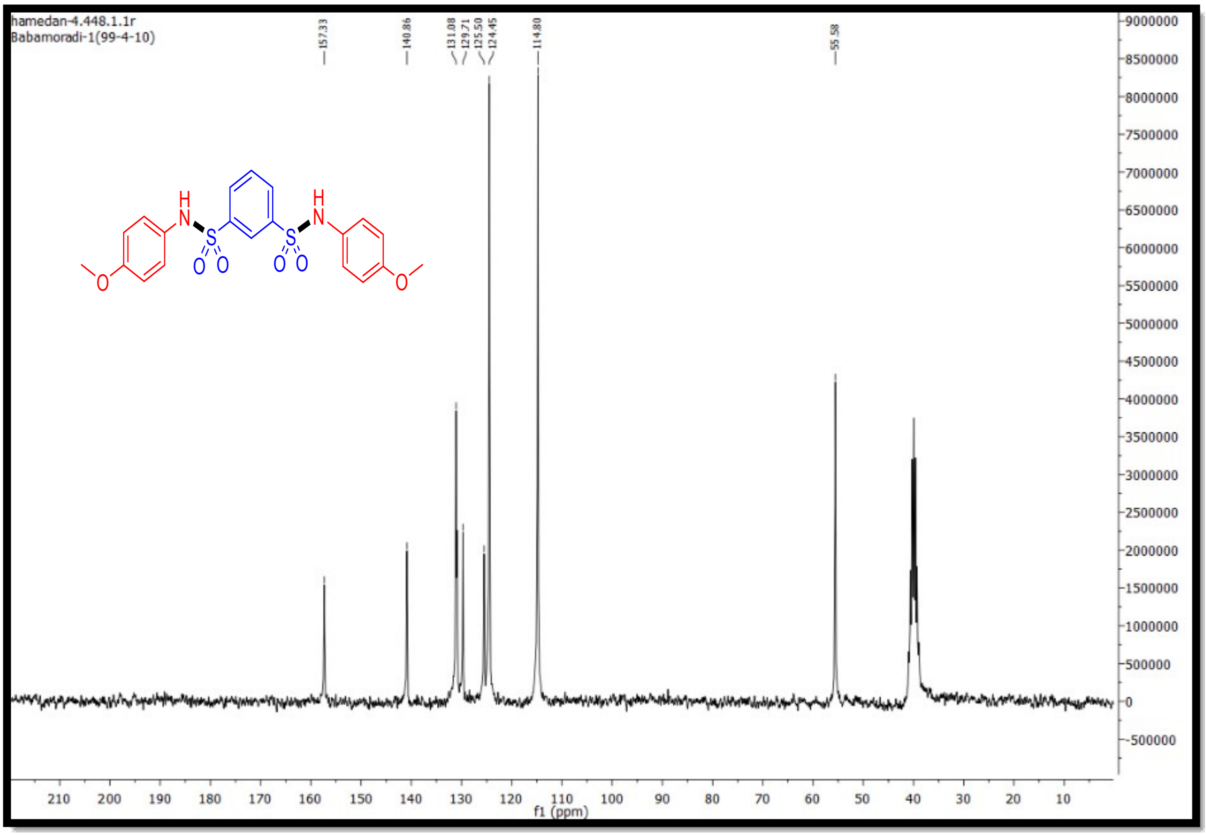
hamedan-4.448.1.1r
Babamoradi-1(99-4-10)
157.33
140.86
131.08
129.71
125.50
124.45
114.80
55.58
f1 (ppm)

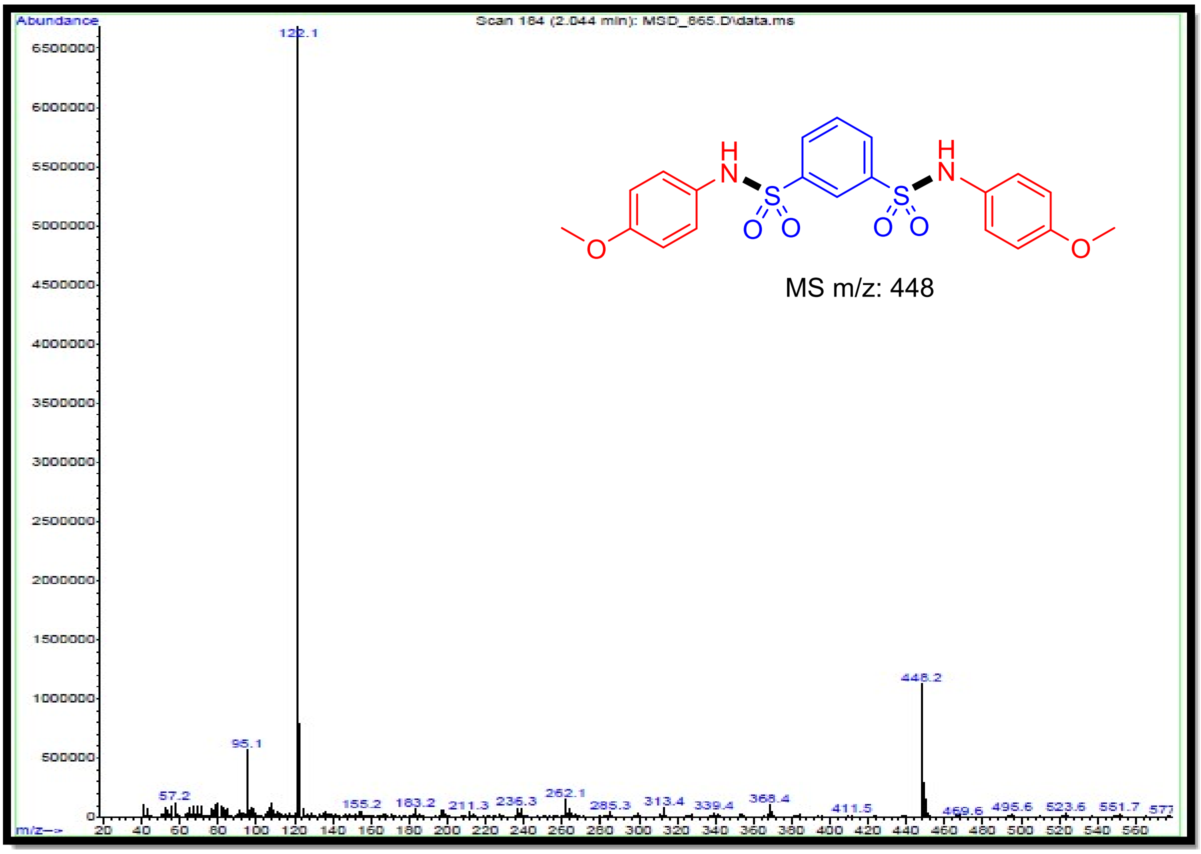
Abundance
Scan 184 (2.044 min): MSD_865.D\data.ms
122.1
95.1
57.2
155.2
183.2
211.3
236.3
262.1
285.3
313.4
339.4
368.4
411.5
448.2
469.6
495.6
523.6
551.7
m/z-->
MS m/z: 448

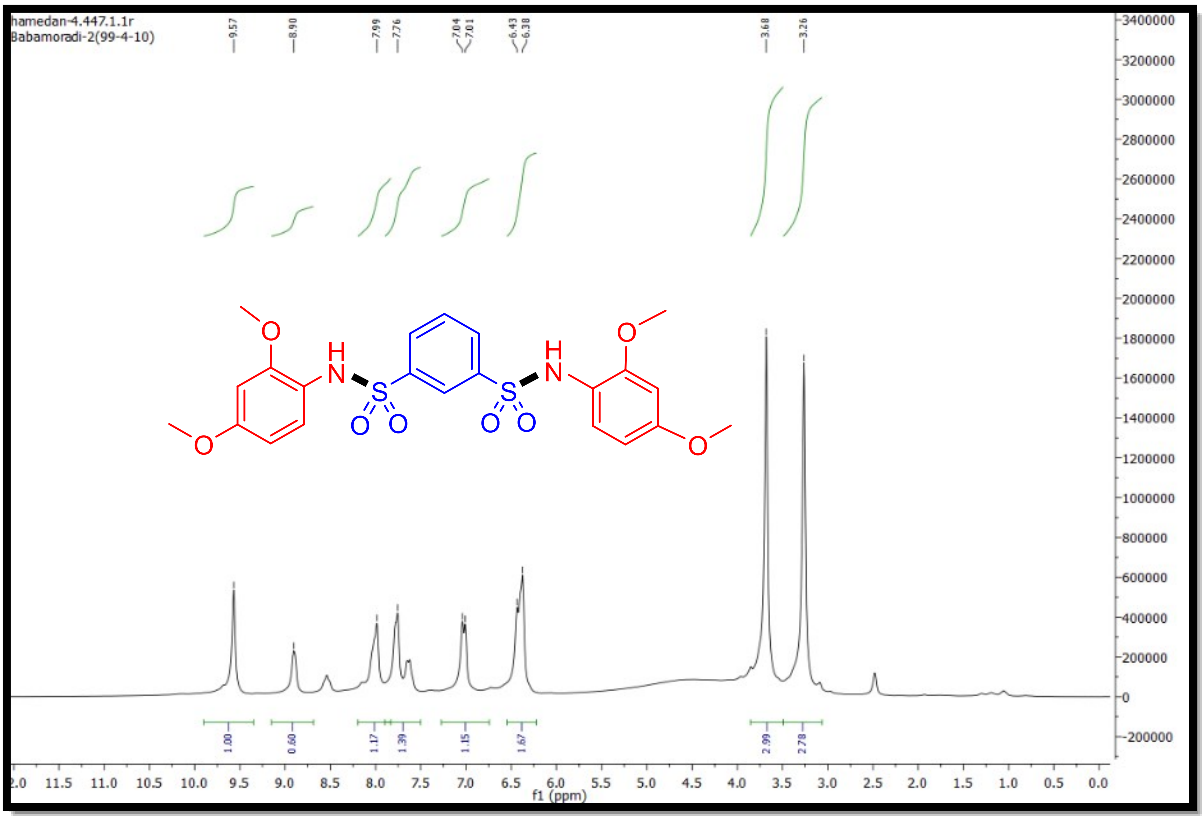
hamedan-4.447.1.1r
Babamoradi-2(99-4-10)
9.57
8.90
7.99
7.76
7.04
7.01
6.43
6.38
3.68
3.26
1.00
0.60
1.17
1.39
1.15
1.67
2.99
2.78
f1 (ppm)

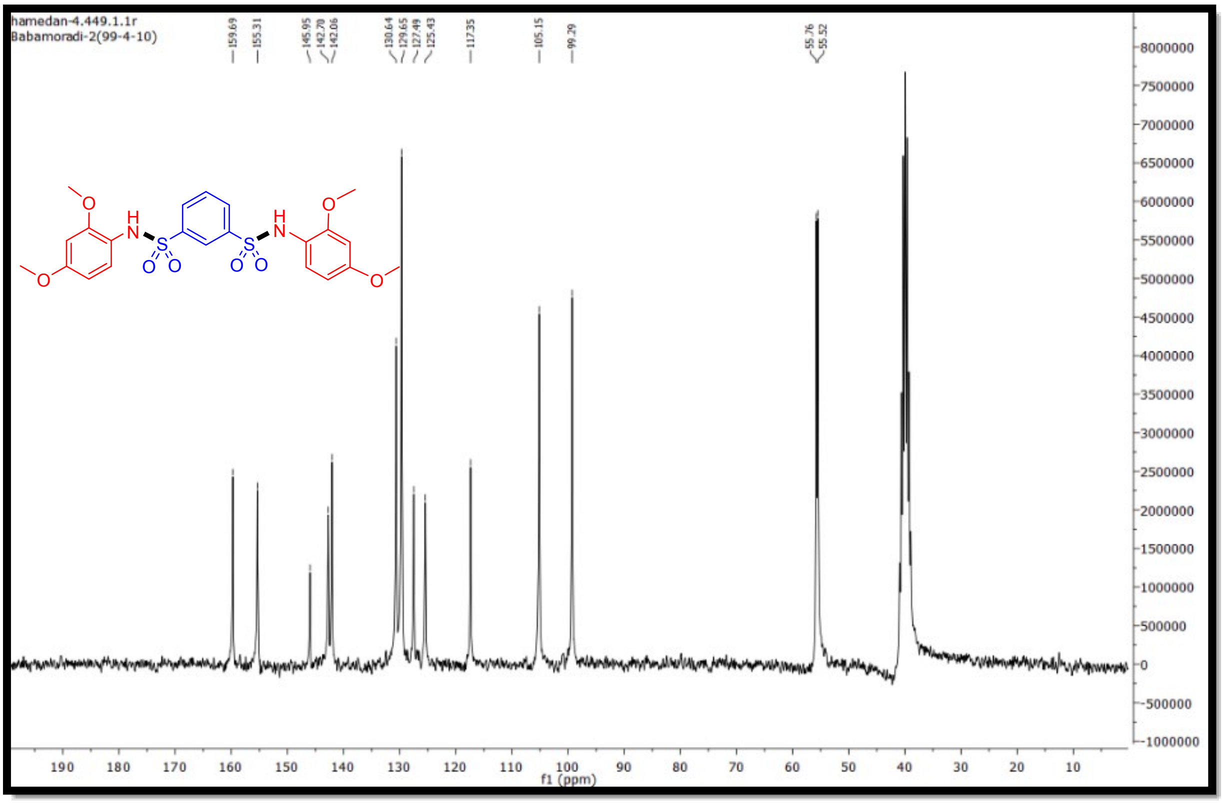
hamedan-4.449.1.1r
Babamoradi-2(99-4-10)
159.69
155.31
145.95
142.70
142.06
130.64
129.65
127.49
125.43
117.35
105.15
99.29
55.76
55.52
f1 (ppm)

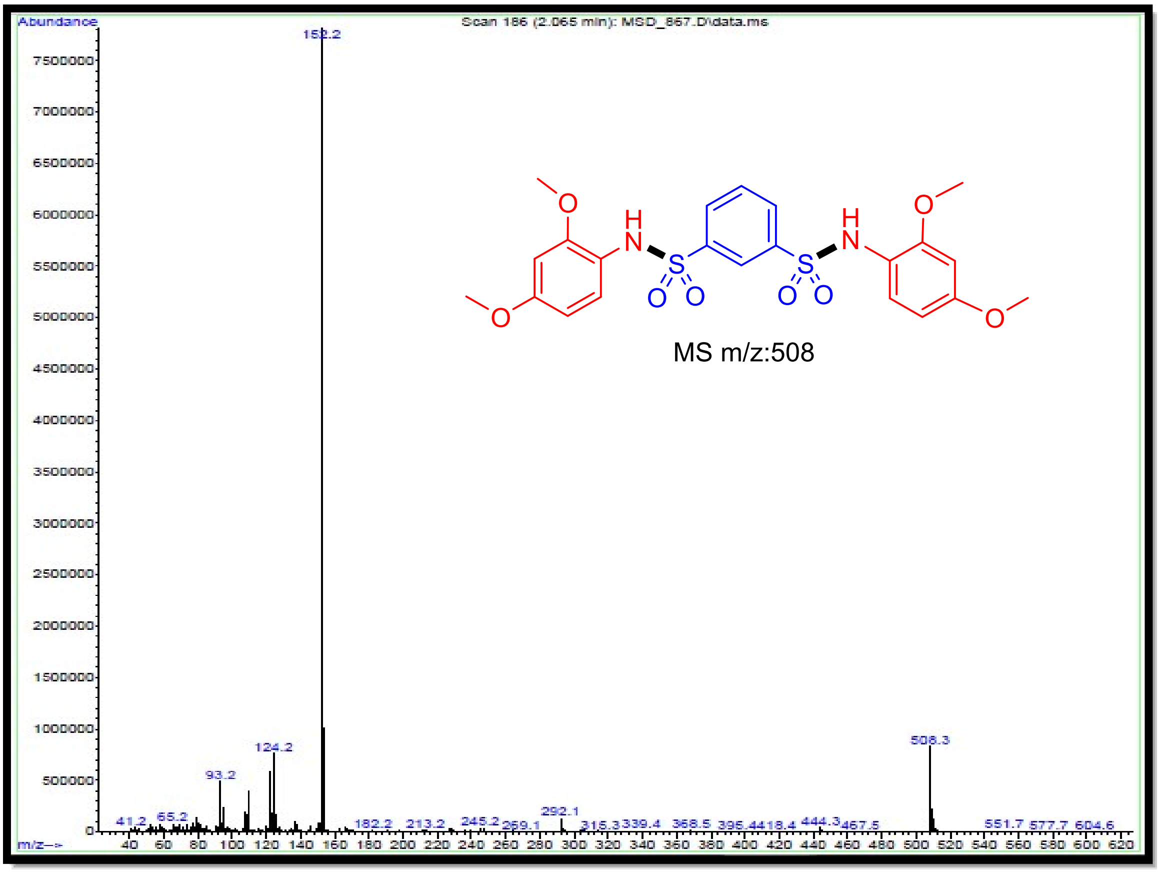
Abundance
Scan 186 (2.065 min): MSD_867.D\data.ms
152.2
124.2
93.2
508.3
292.1
m/z-->
MS m/z:508

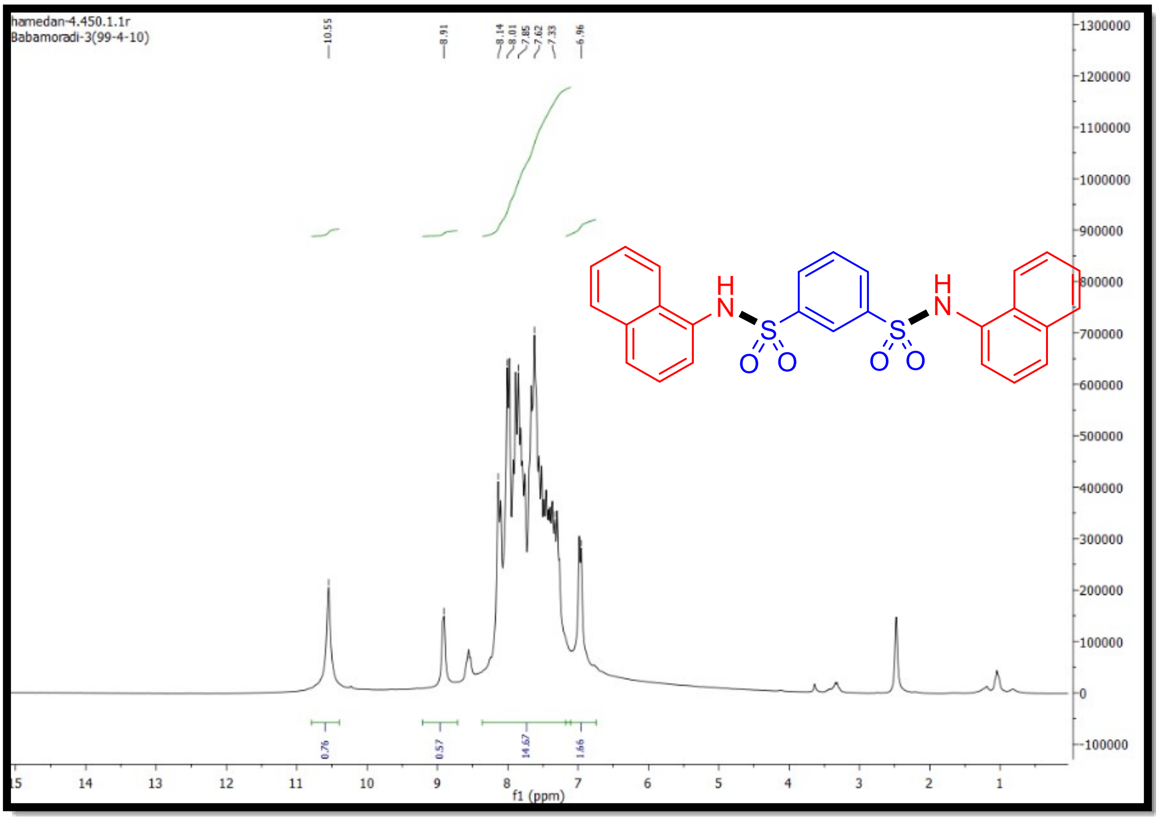
hamedan-4.450.1.1r
Babamoradi-3(99-4-10)
10.55
8.91
8.14
8.01
7.85
7.62
7.33
6.96
0.76
0.57
14.67
1.66
f1 (ppm)

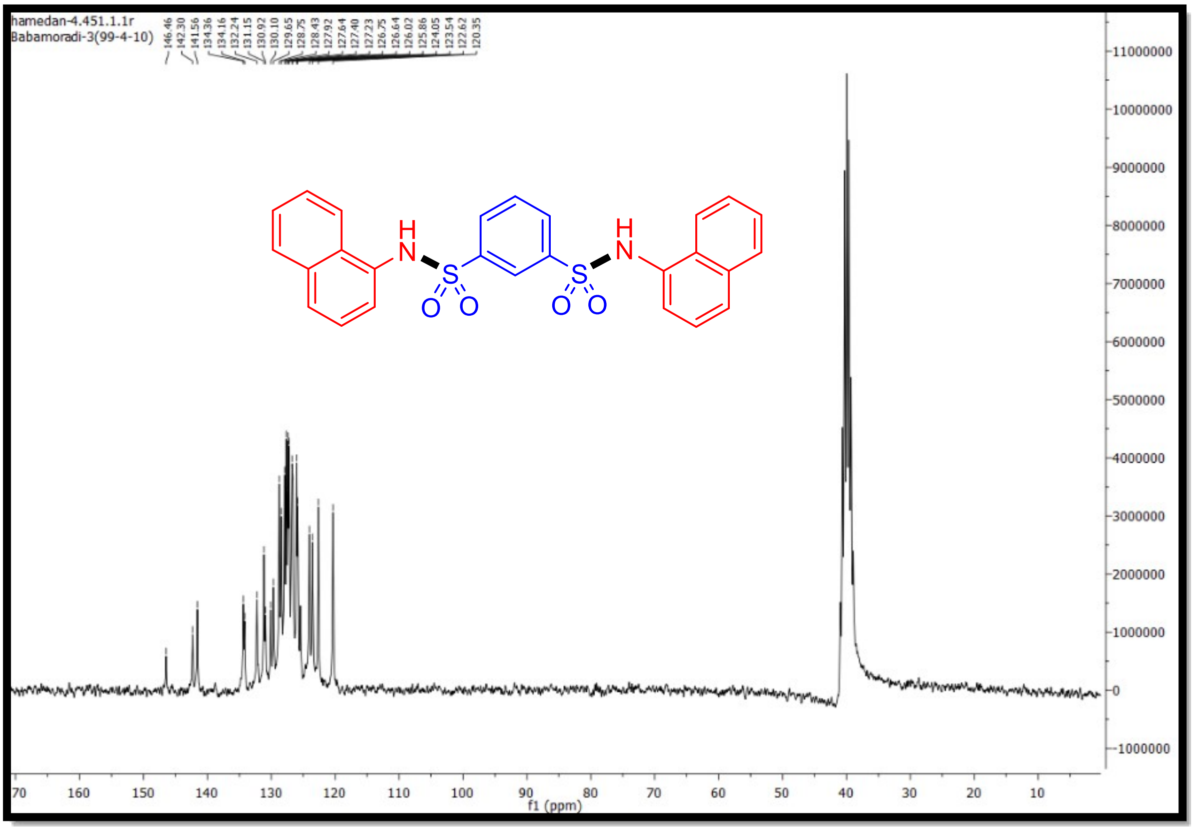
hamedan-4.451.1.1r
Babamoradi-3(99-4-10)
146.46
142.30
141.56
134.36
134.16
132.24
131.15
130.92
130.10
129.65
128.75
128.43
127.92
127.64
127.40
127.23
126.75
126.64
126.02
125.86
124.05
123.54
122.62
120.35
f1 (ppm)

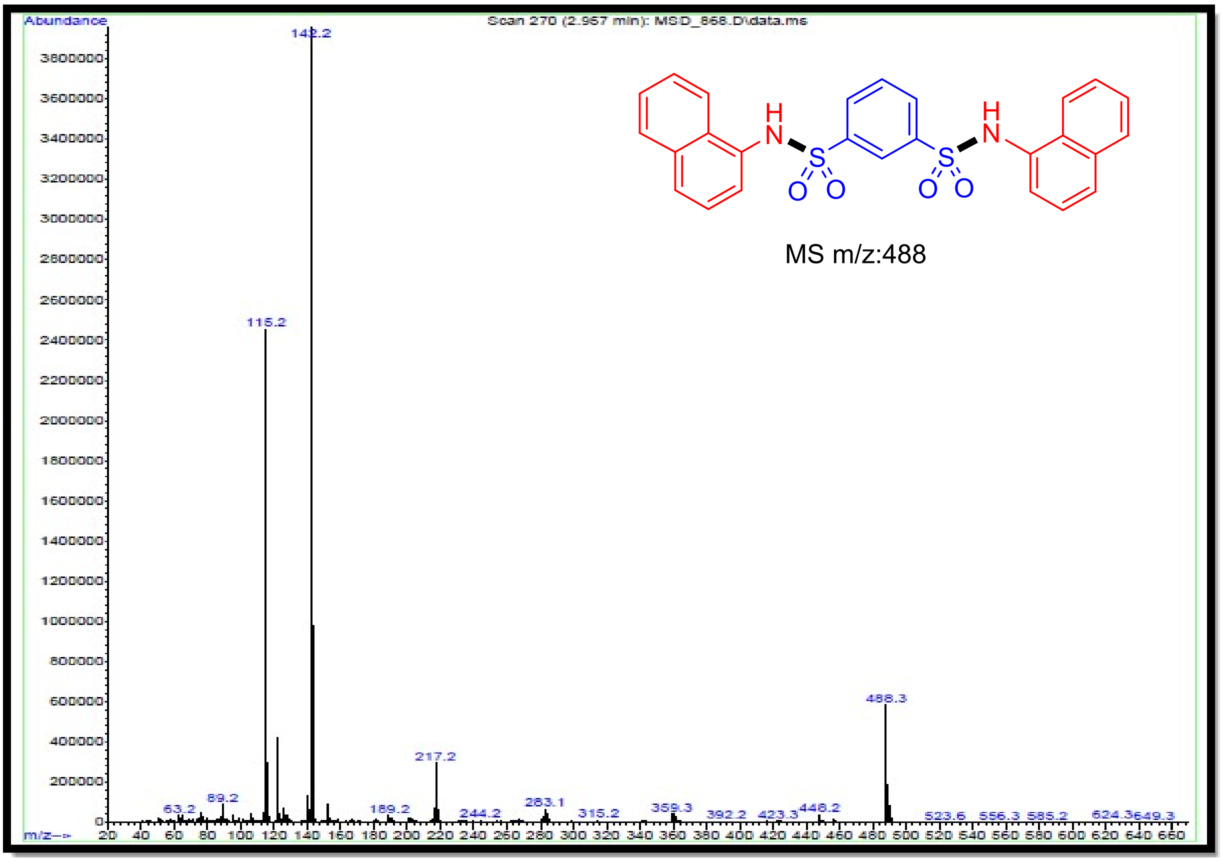
Abundance
Scan 270 (2.957 min): MSD_868.D\data.ms
142.2
115.2
217.2
488.3
MS m/z:488
m/z-->

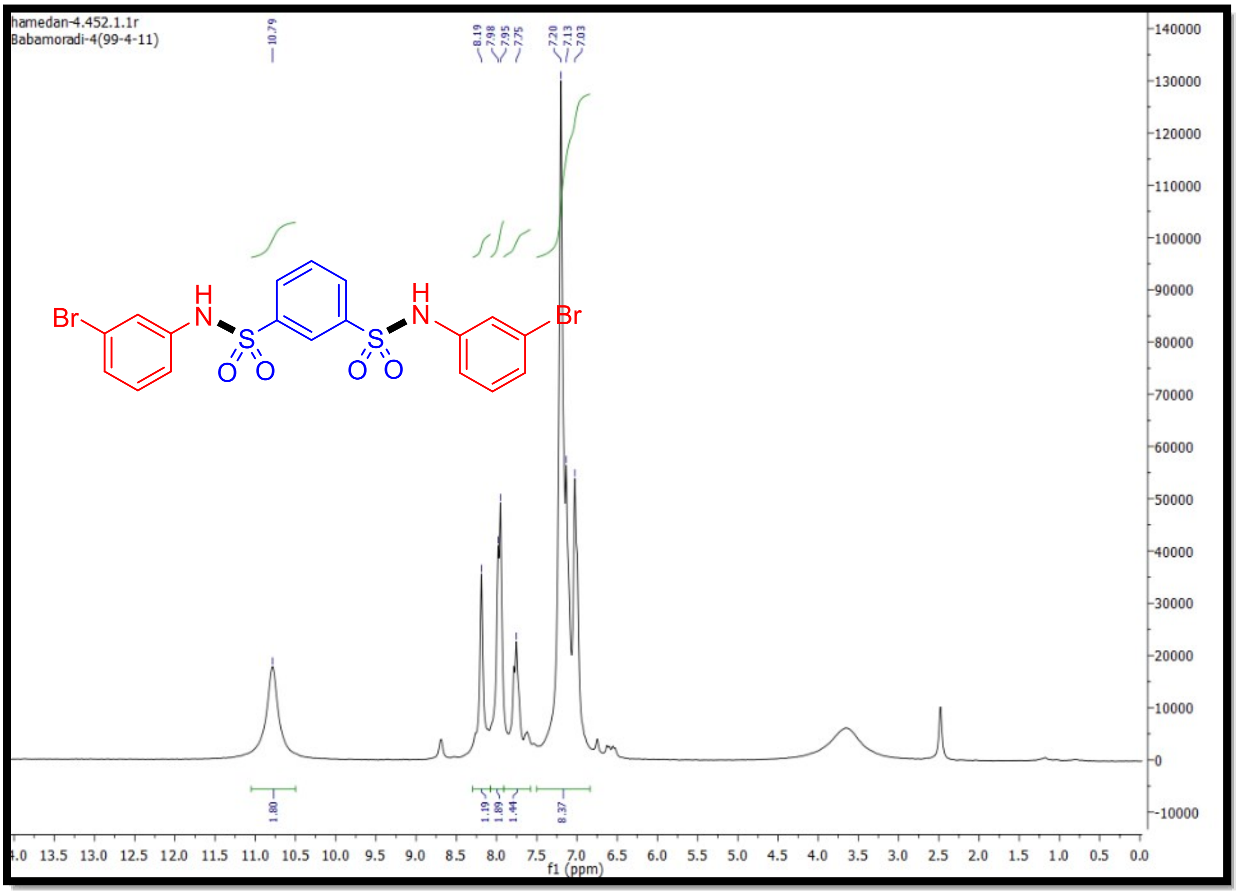
hamedan-4.452.1.1r
Babamoradi-4(99-4-11)
10.79
8.19
7.98
7.95
7.75
7.20
7.13
7.03
1.80
1.19
1.89
1.44
8.37
f1 (ppm)
Br
H
N
S
O
O
H
N
S
O
O
Br

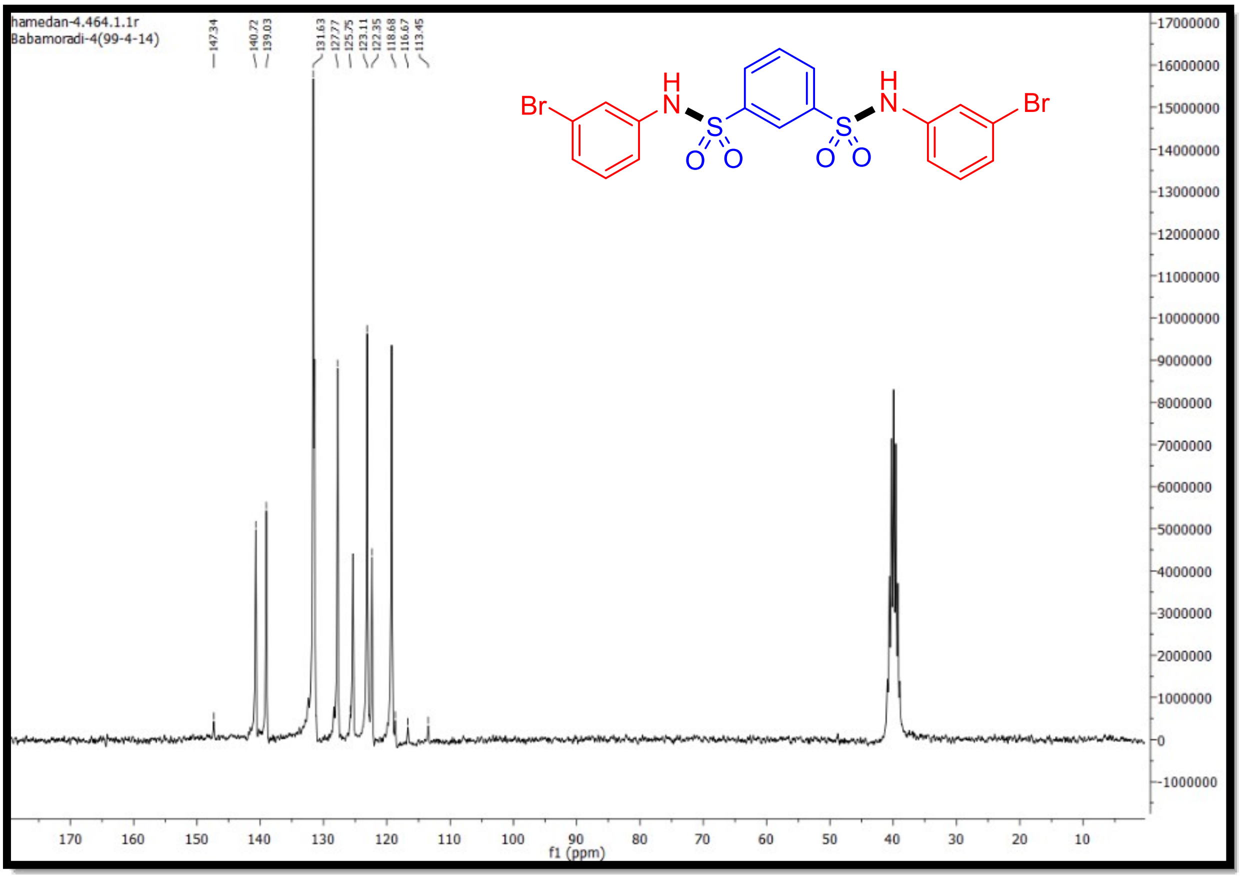
hamedan-4.464.1.1r
Babamoradi-4(99-4-14)
147.34
140.72
139.03
131.63
127.77
125.75
123.11
122.35
118.68
116.67
113.45
f1 (ppm)
Br
H
N
S
O
O
H
N
S
O
O
Br

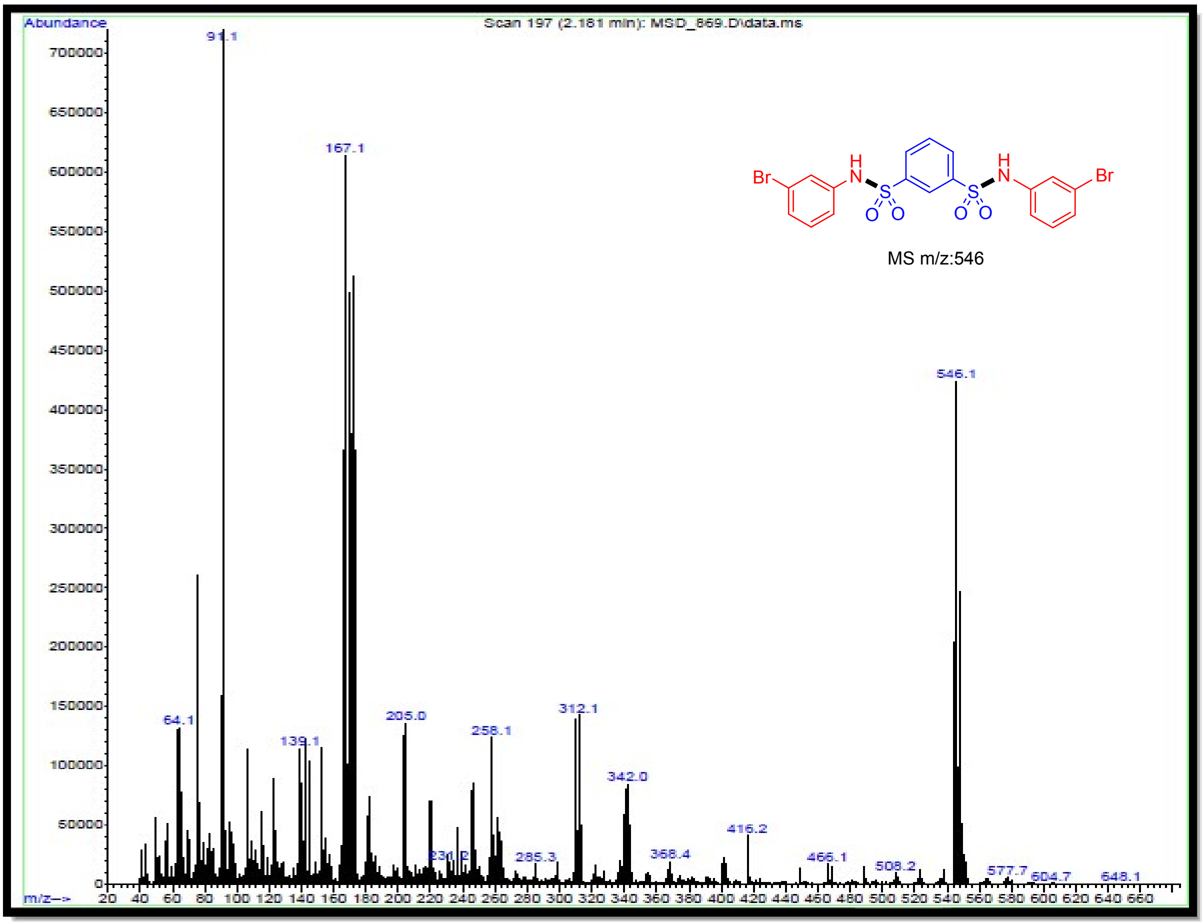

Abundance
Scan 197 (2.181 min): MSD_869.D\data.ms
m/z-->
MS m/z:546

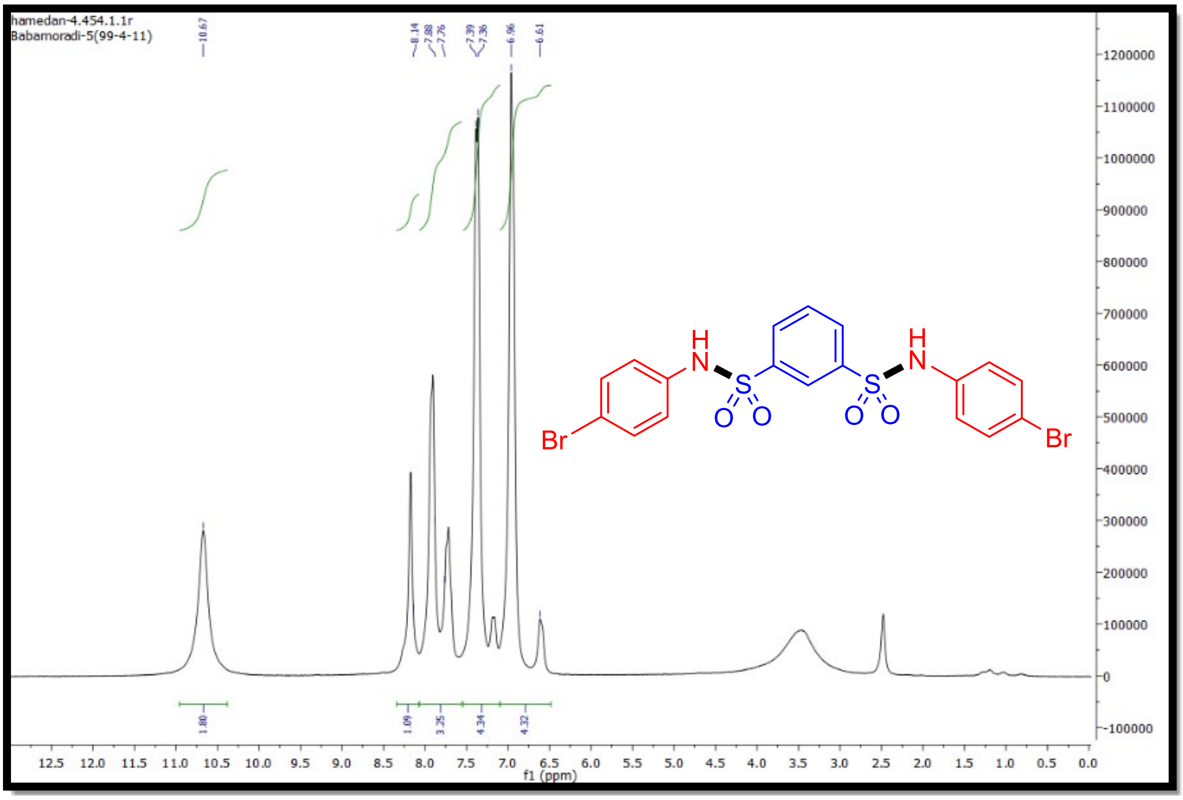

hamedan-4.454.1.1r
Babamoradi-5(99-4-11)
f1 (ppm)

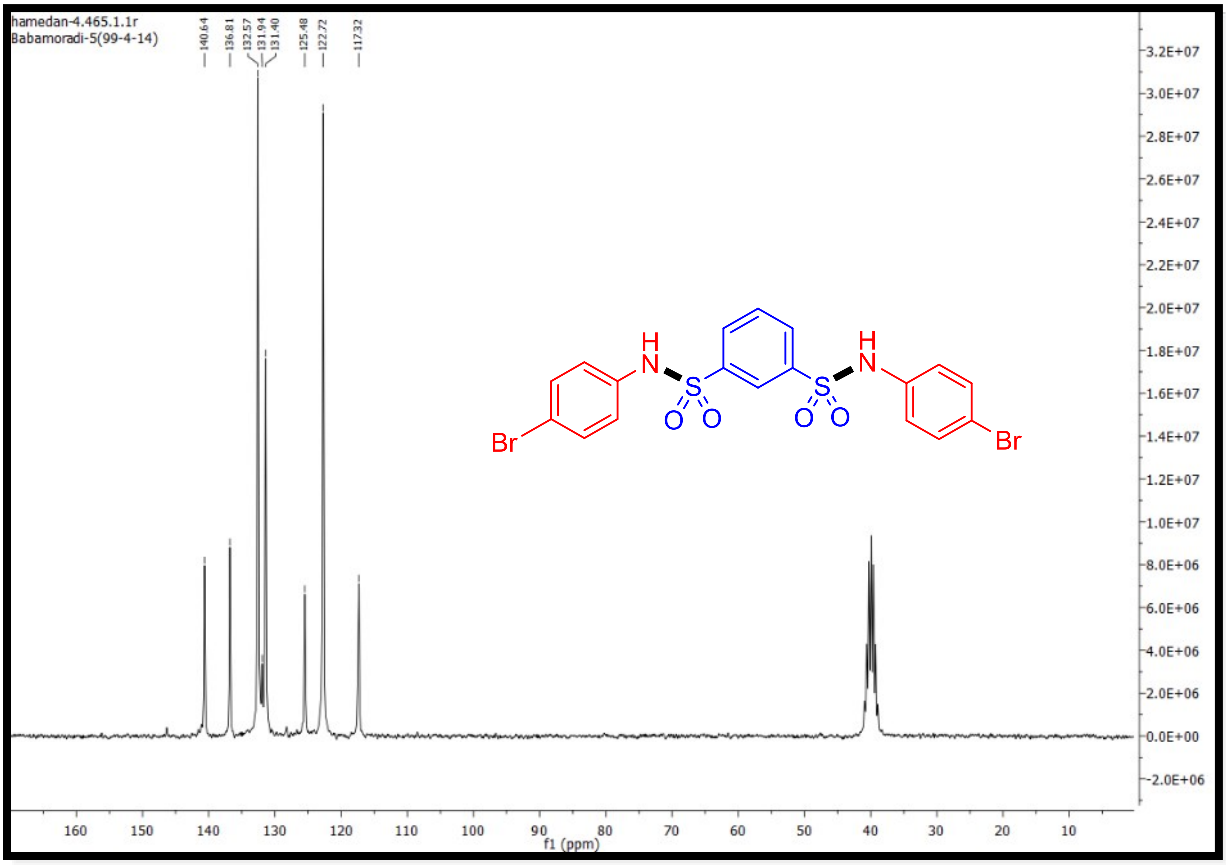
hamedan-4.465.1.1r
Babamoradi-5(99-4-14)
140.64
136.81
132.57
131.94
131.40
125.48
122.72
117.32
H
N
S
O
O
Br
f1 (ppm)

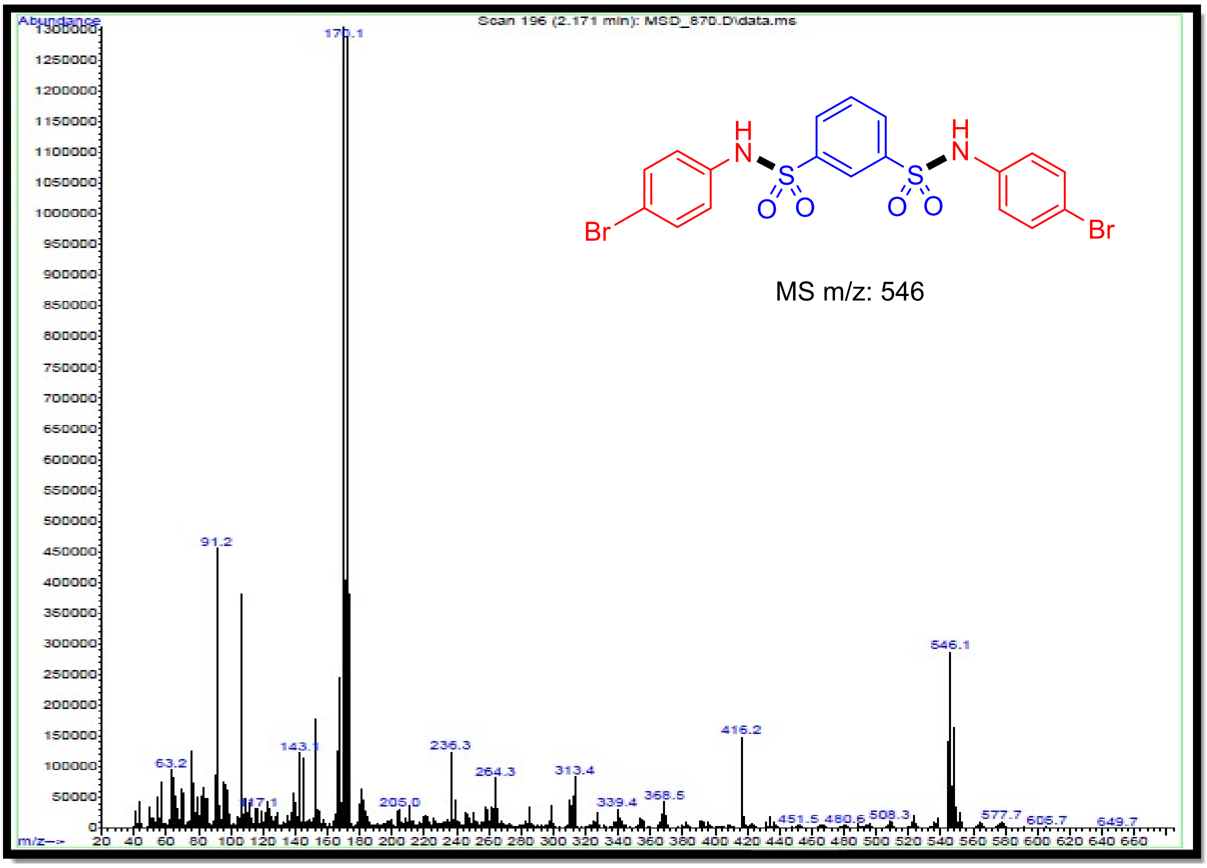
Abundance
Scan 196 (2.171 min): MSD_870.D\data.ms
170.1
91.2
63.2
143.1
117.1
205.0
236.3
264.3
313.4
339.4
368.5
416.2
451.5
480.6
508.3
546.1
577.7
605.7
649.7
m/z-->
H
N
S
O
O
Br
MS m/z: 546

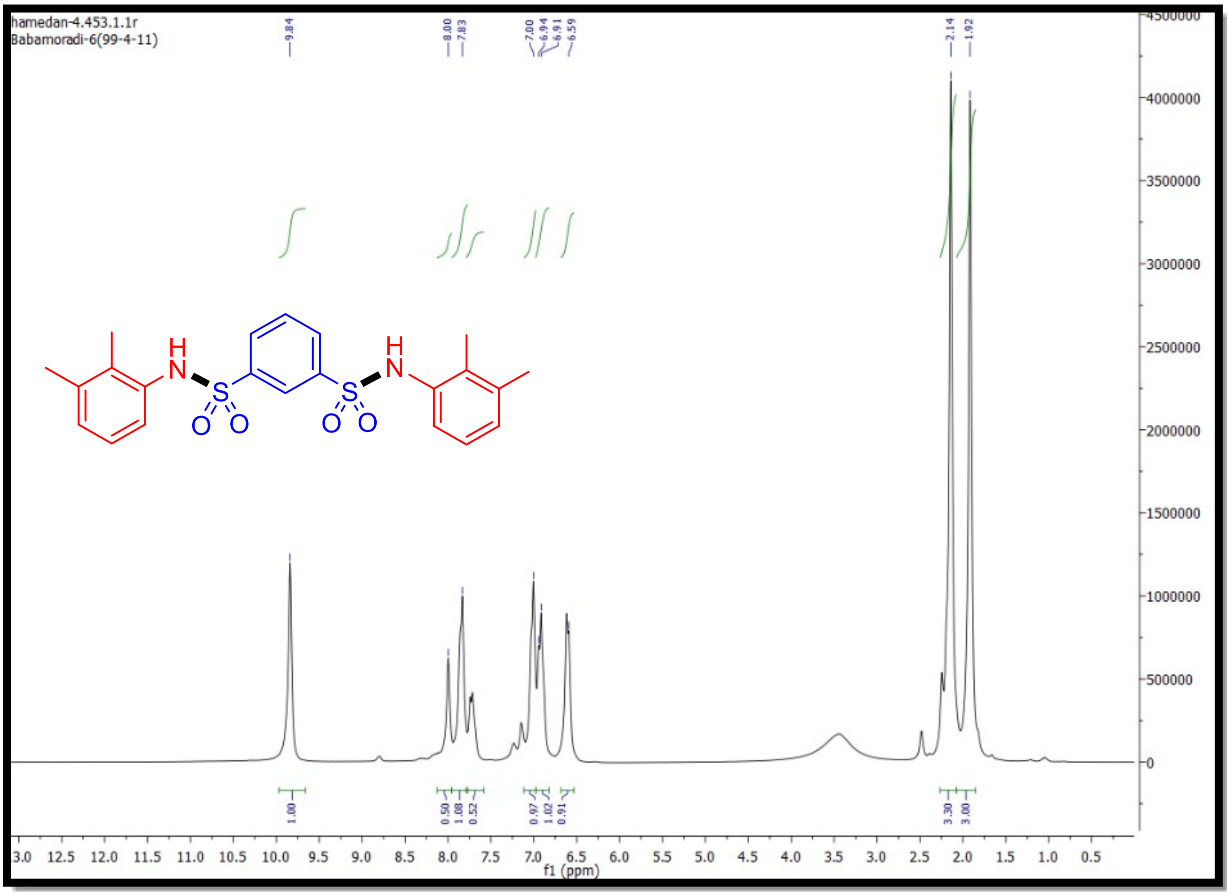

hamedan-4.453.1.1r
Babamoradi-6(99-4-11)
f1 (ppm)

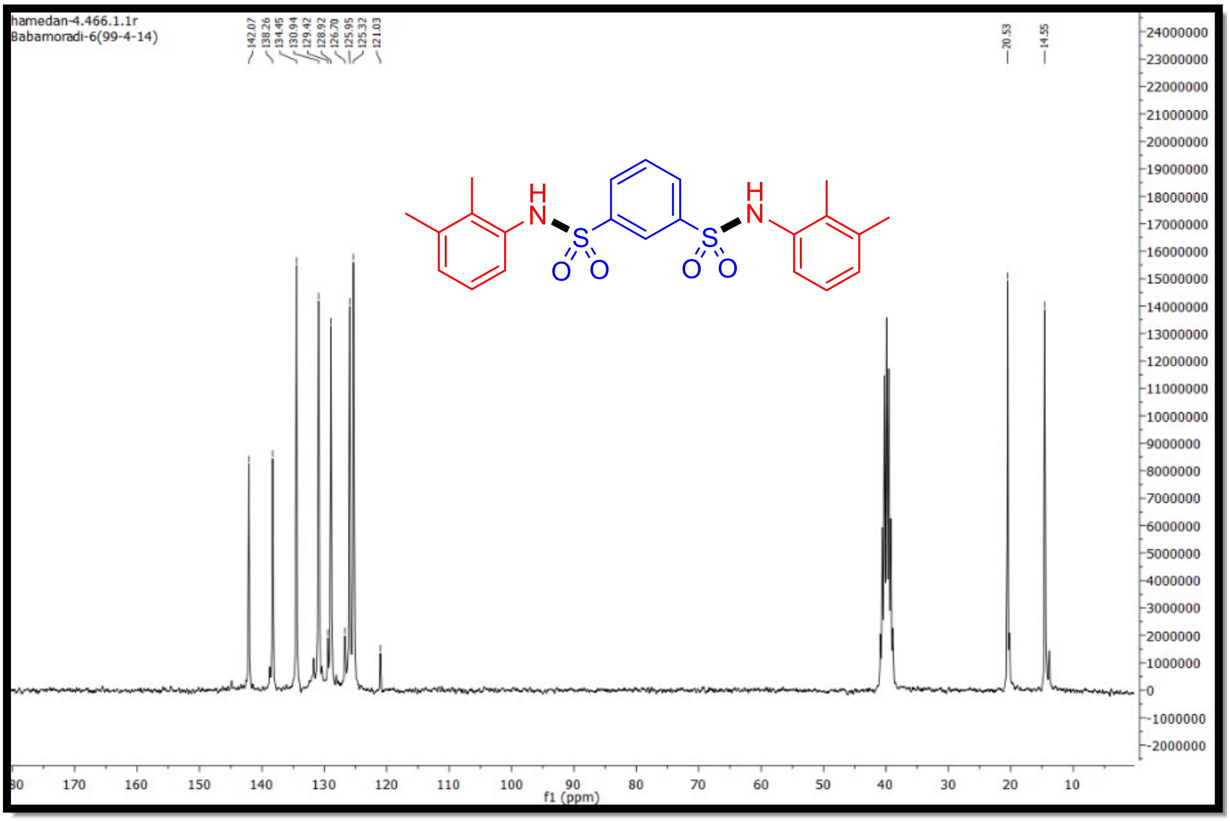

hamedan-4.466.1.1r
Babamoradi-6(99-4-14)
f1 (ppm)

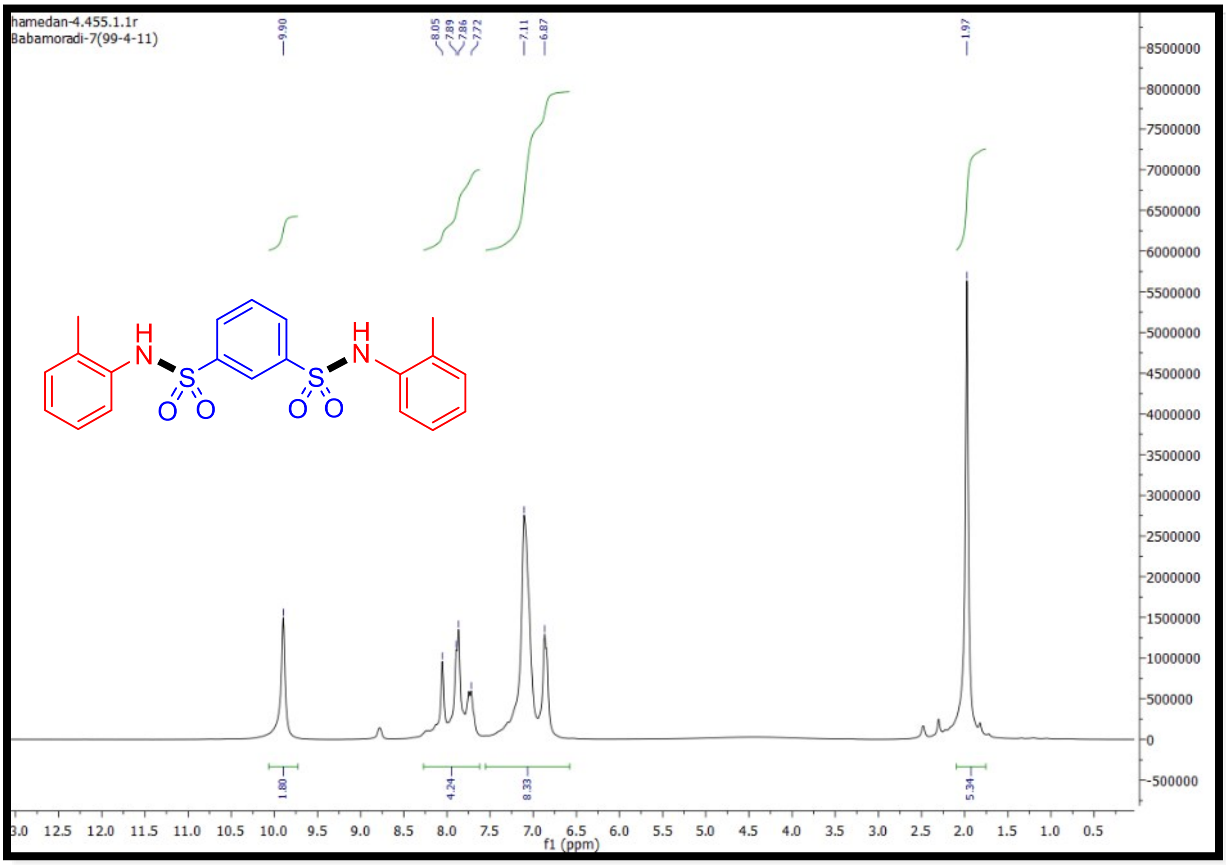
hamedan-4.455.1.1r
Babamoradi-7(99-4-11)
9.90
8.05
7.89
7.86
7.72
7.11
6.87
1.97
1.80
4.24
8.33
5.34
f1 (ppm)

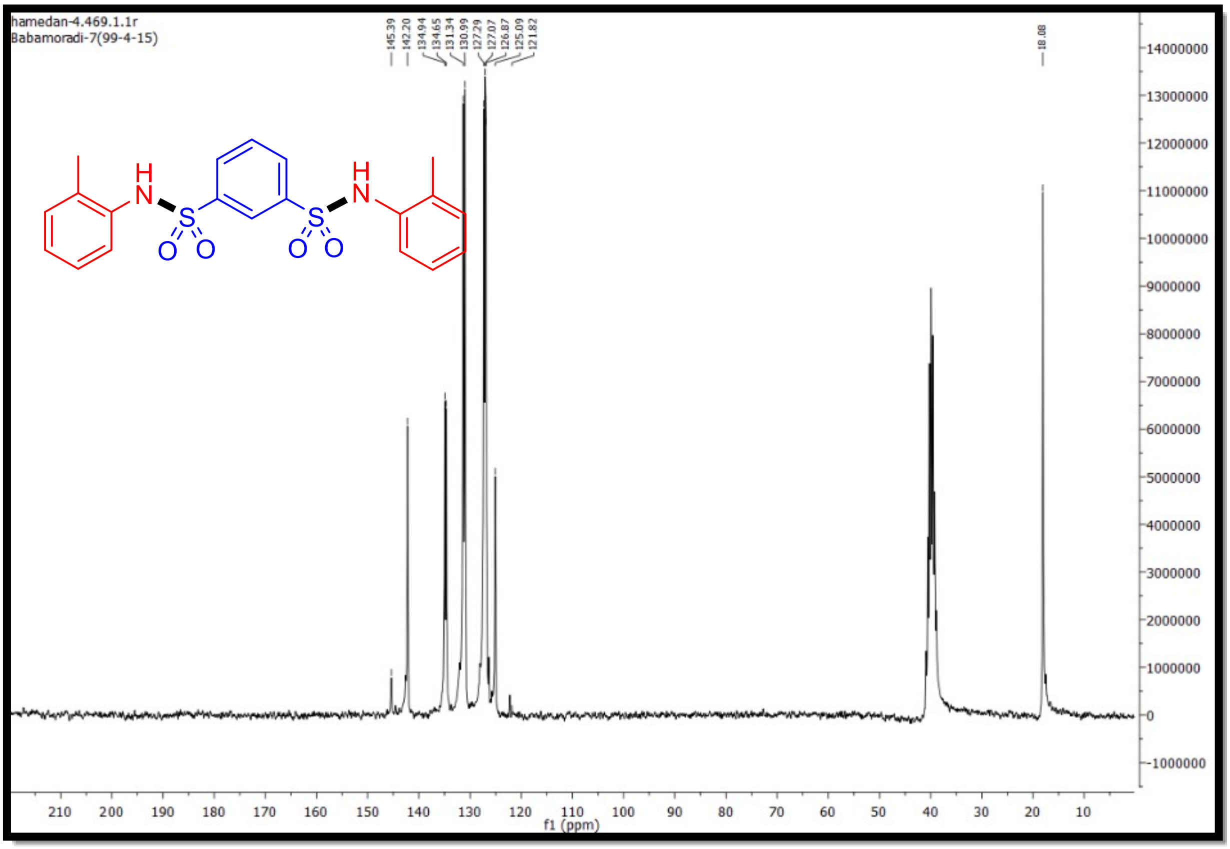
hamedan-4.469.1.1r
Babamoradi-7(99-4-15)
145.39
142.20
134.94
134.65
131.34
130.99
127.29
127.07
126.87
125.09
121.82
18.08
f1 (ppm)

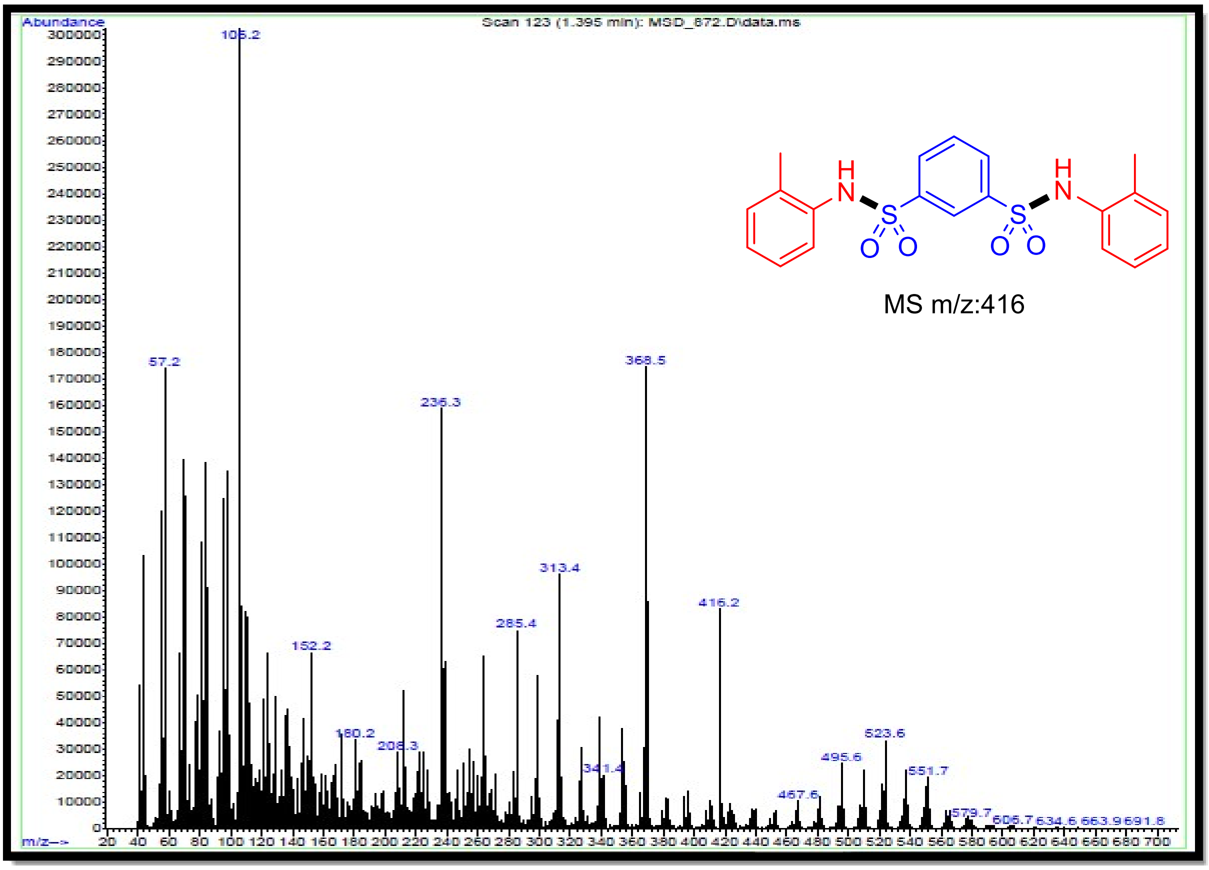
Abundance
Scan 123 (1.395 min): MSD_872.D\data.ms
105.2
57.2
368.5
236.3
313.4
285.4
416.2
152.2
180.2
206.3
341.4
523.6
495.6
551.7
467.6
579.7
605.7
634.6
663.9
691.8
m/z-->
MS m/z:416

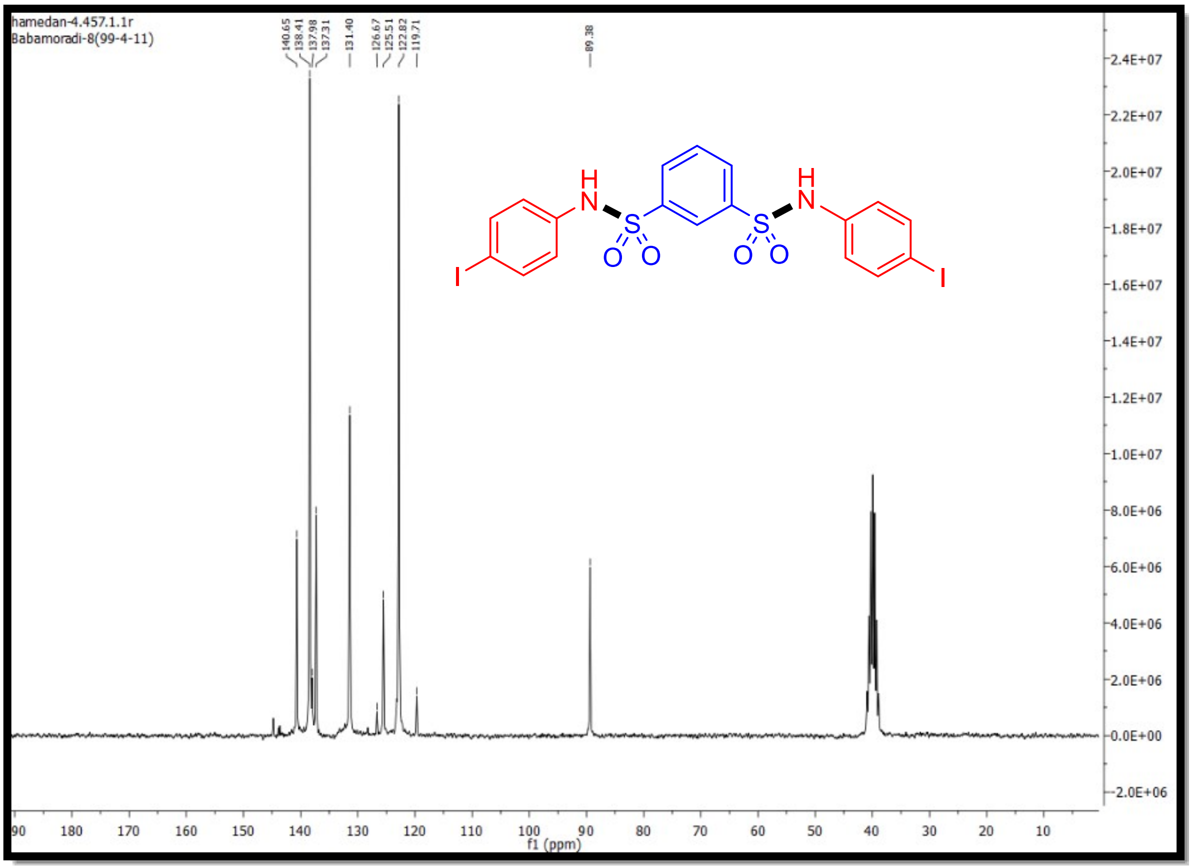
hamedan-4.457.1.1r
Babamoradi-8(99-4-11)
140.65
138.41
137.98
137.31
131.40
126.67
125.51
122.82
119.71
89.38
f1 (ppm)

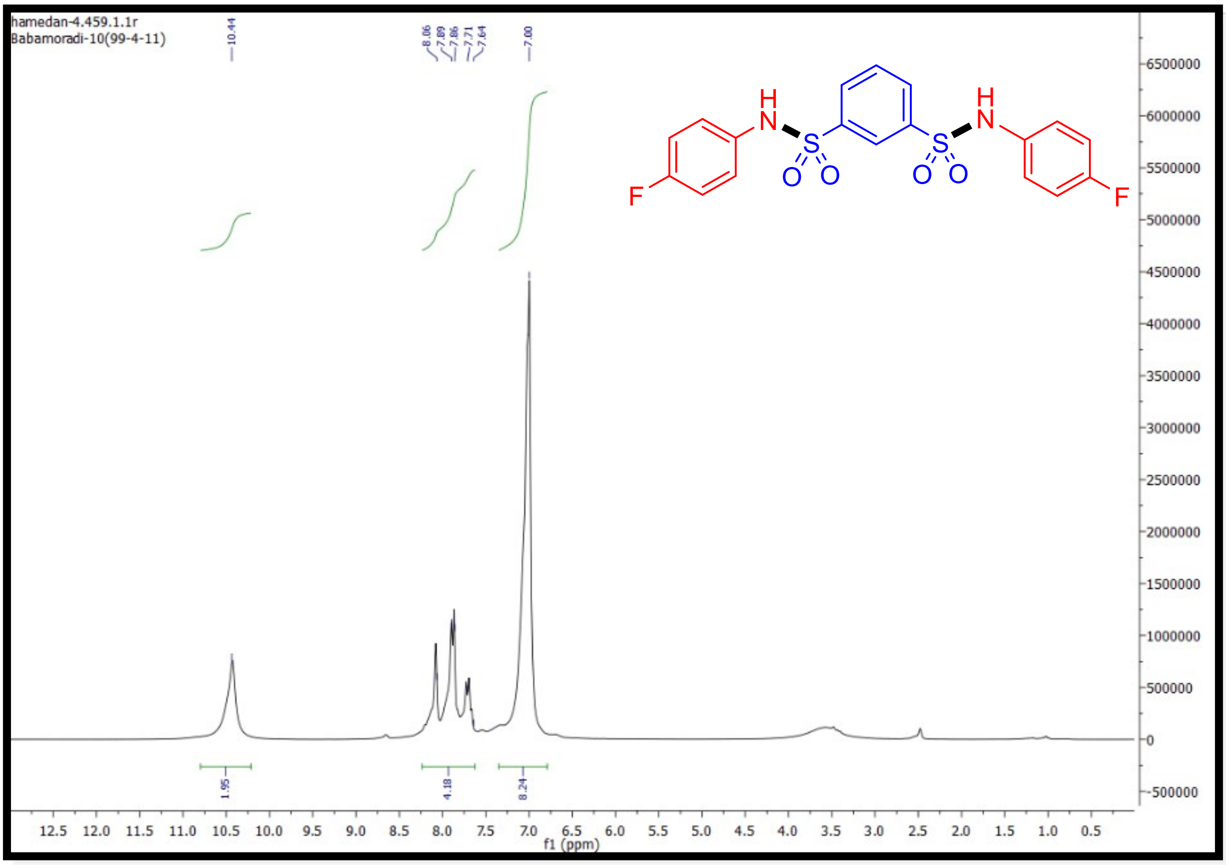
hamedan-4.459.1.1r
Babamoradi-10(99-4-11)
10.44
8.06
7.89
7.86
7.71
7.64
7.00
1.95
4.18
8.24
f1 (ppm)

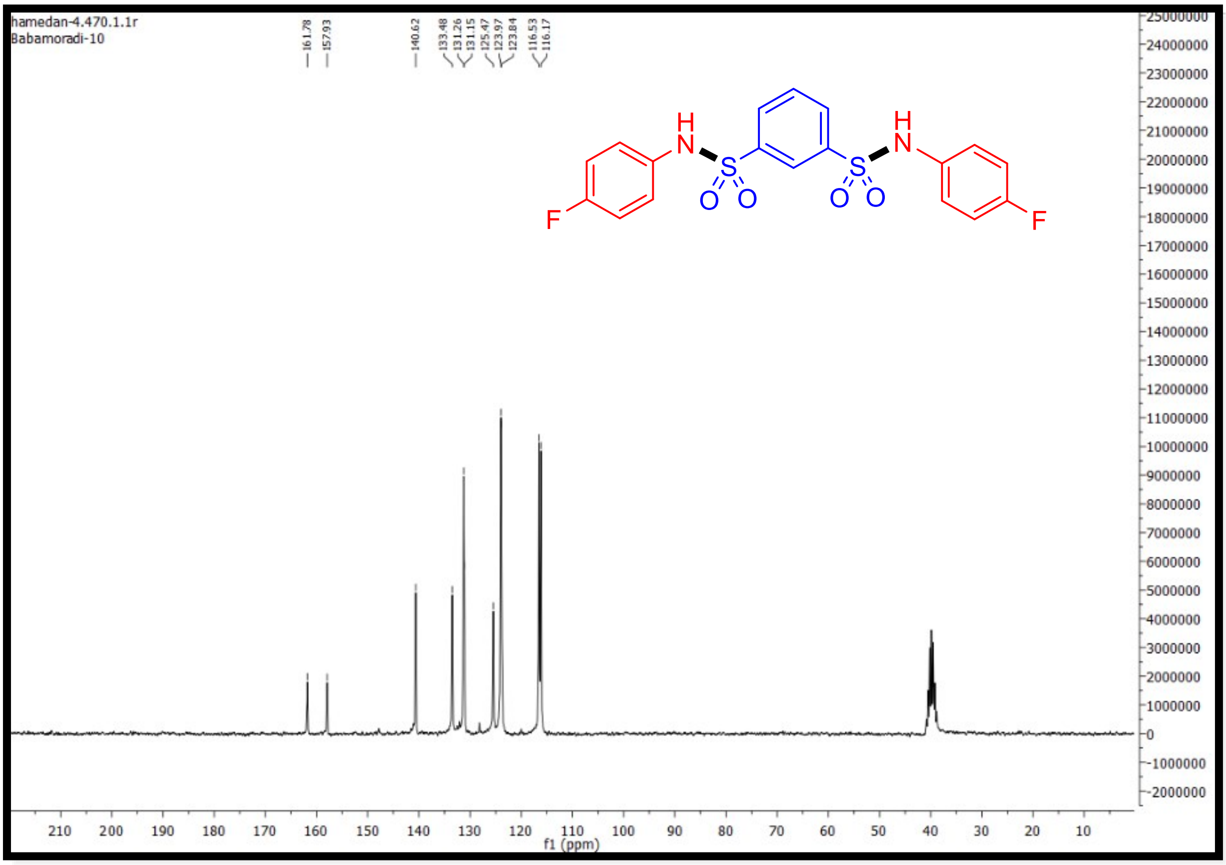
hamedan-4.470.1.1r
Babamoradi-10
161.78
157.93
140.62
133.48
131.26
131.15
125.47
123.97
123.84
116.53
116.17
f1 (ppm)

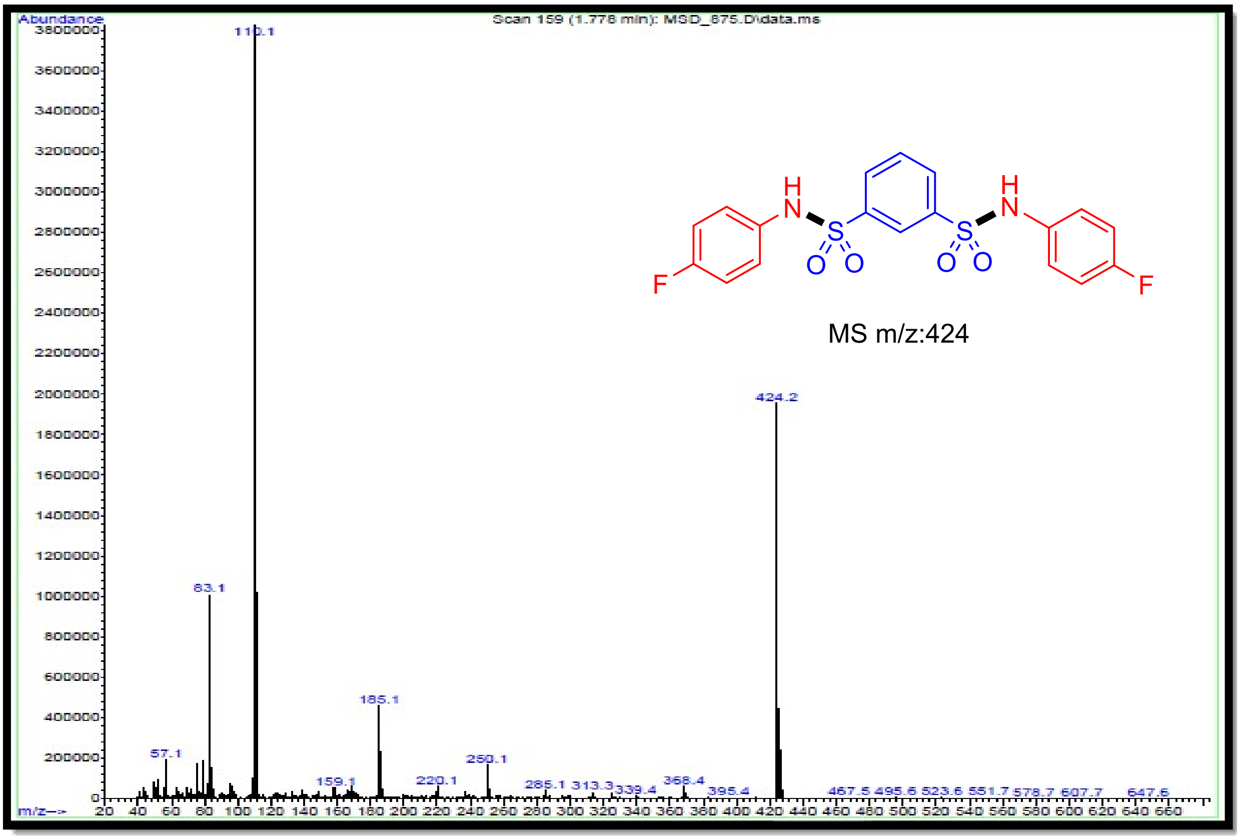
Abundance
Scan 159 (1.776 min): MSD_875.D\data.ms
110.1
424.2
83.1
185.1
57.1
250.1
MS m/z:424
m/z-->

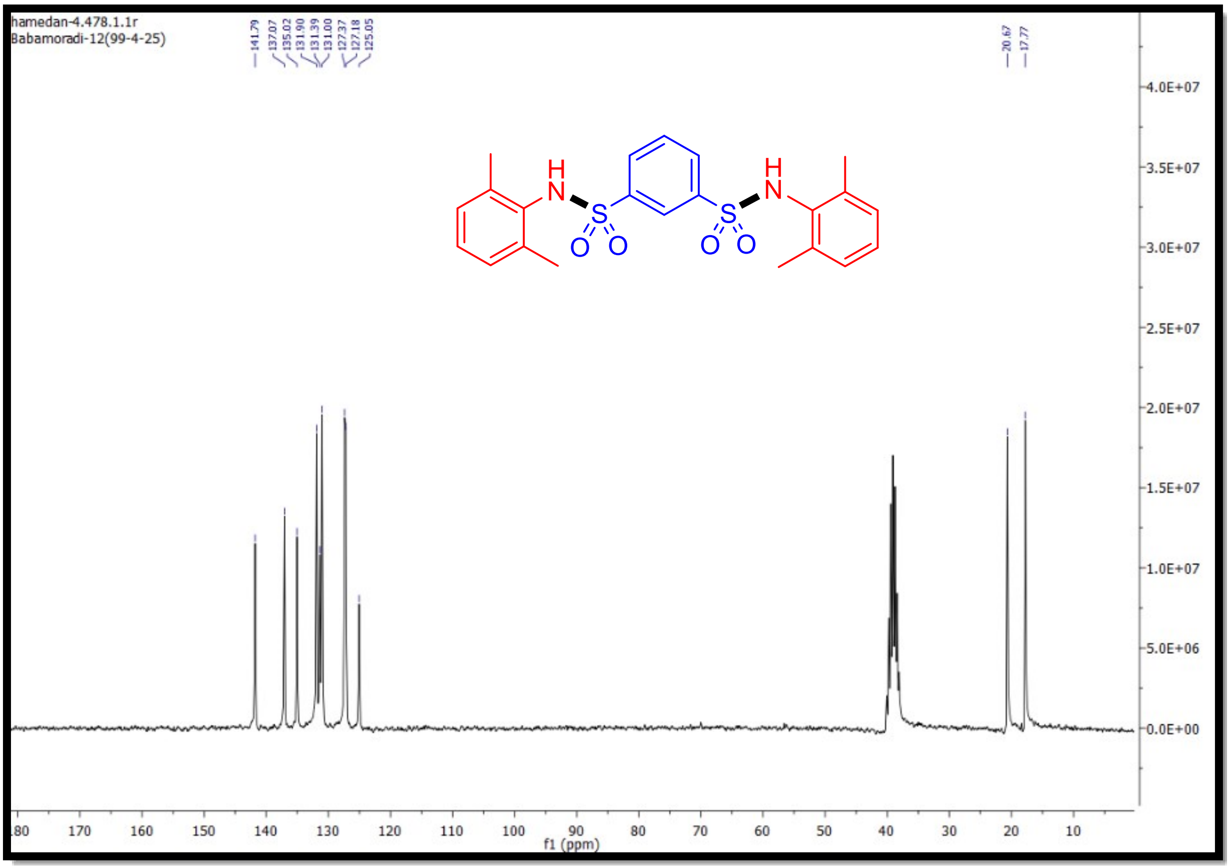
hamedan-4.478.1.1r
Babamoradi-12(99-4-25)
141.79
137.07
135.02
131.90
131.39
131.00
127.37
127.18
125.05
20.67
17.77
f1 (ppm)

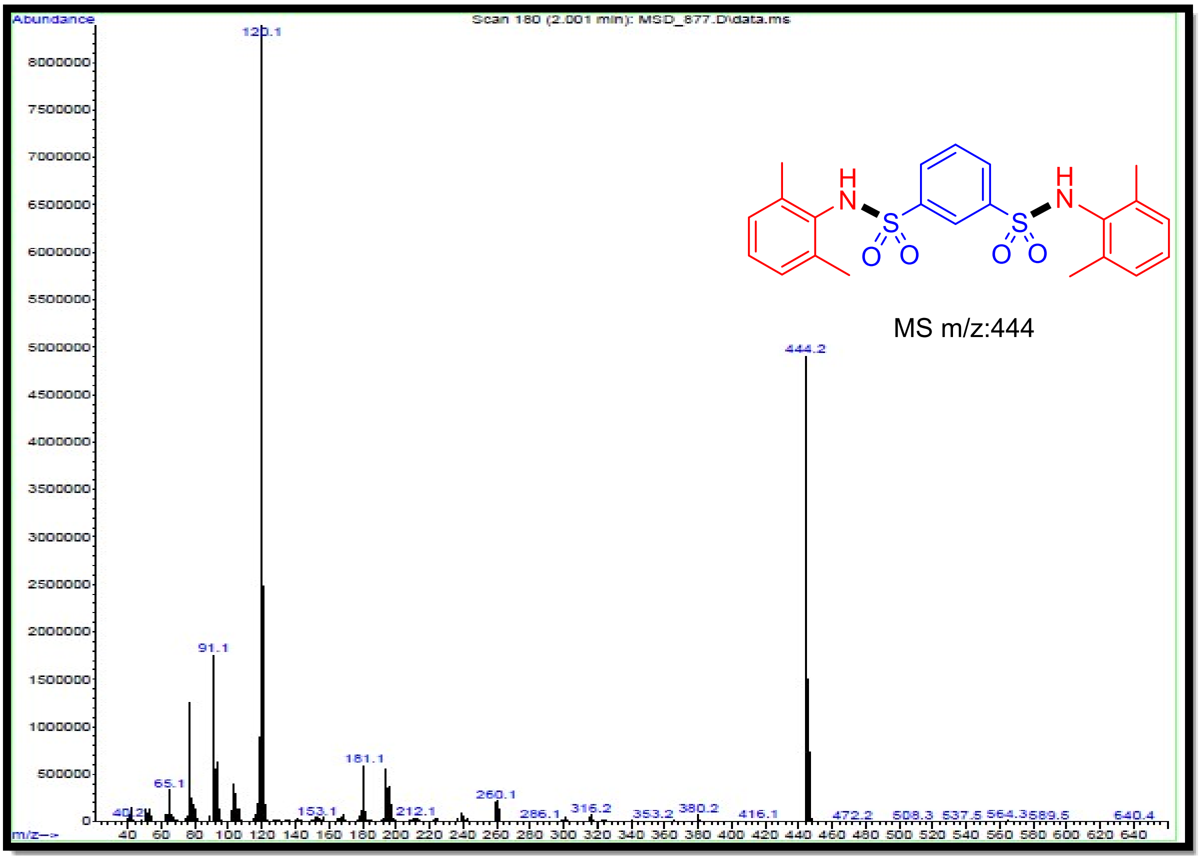

Abundance
Scan 180 (2.001 min): MSD_877.D\data.ms
120.1
91.1
65.1
181.1
260.1
444.2
m/z-->
MS m/z:444

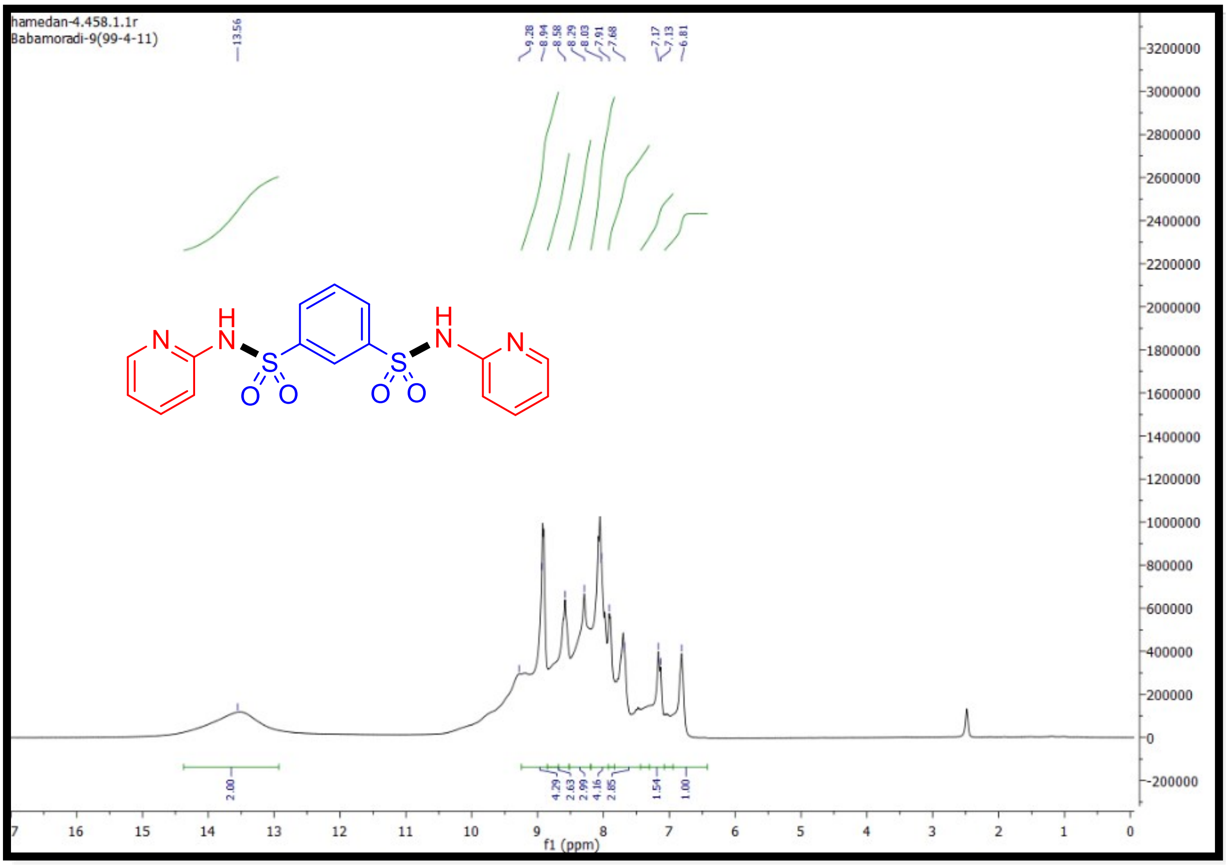

hamedan-4.458.1.1r
Babamoradi-9(99-4-11)
13.56
9.28
8.94
8.58
8.29
8.03
7.91
7.68
7.17
7.13
6.81
f1 (ppm)

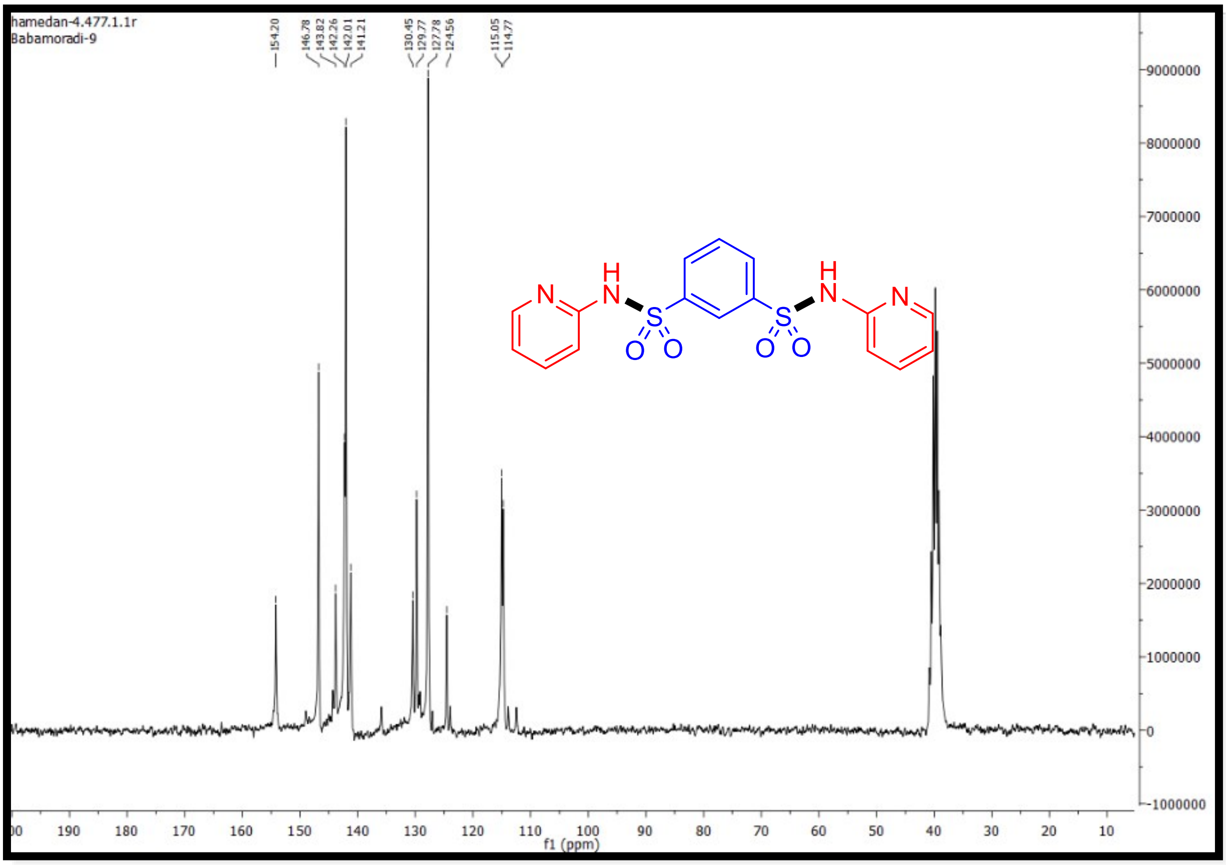
hamedan-4.477.1.1r
Babamoradi-9
154.20
146.78
143.82
142.26
142.01
141.21
130.45
129.77
127.78
124.56
115.05
114.77
f1 (ppm)

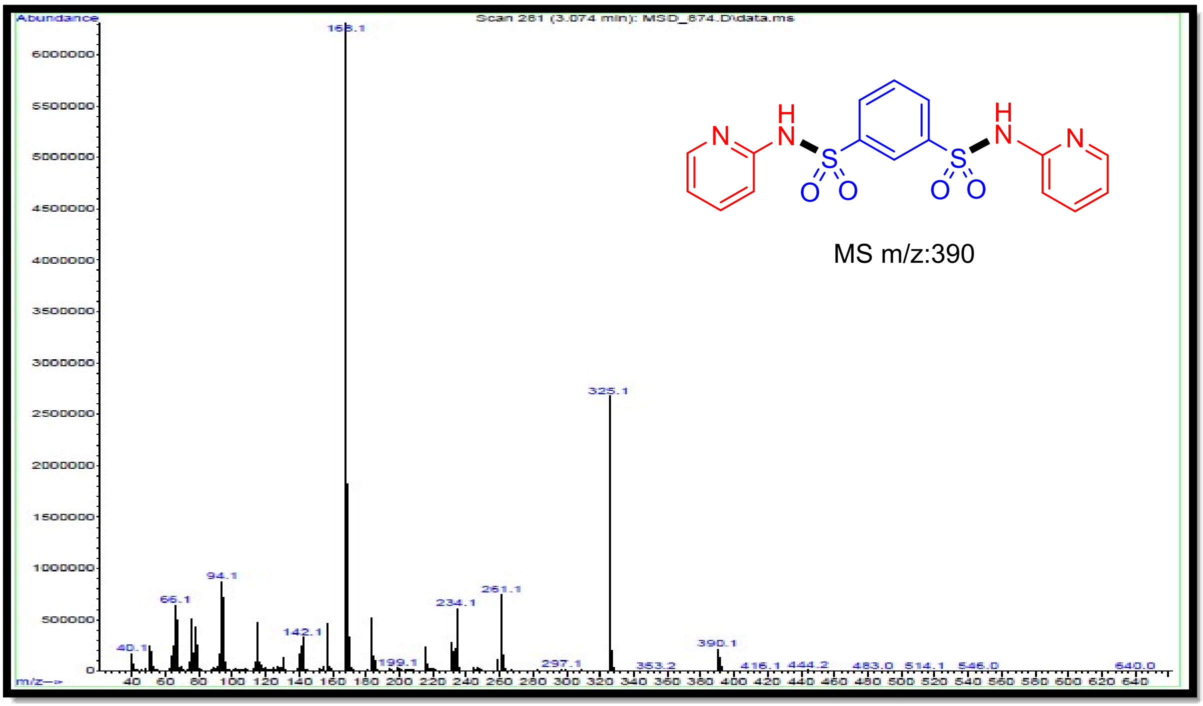
Abundance
Scan 281 (3.074 min): MSD_874.D\data.ms
325.1
94.1
66.1
40.1
142.1
199.1
234.1
261.1
297.1
353.2
390.1
416.1
444.2
483.0
514.1
546.0
640.0
m/z-->
MS m/z:390
